# Benchmark Investigation of SARS-CoV-2 Mutants’ Immune Escape with 2B04 Murine Antibody: A Step Towards Unraveling a Larger Picture

**DOI:** 10.3390/cimb46110745

**Published:** 2024-11-06

**Authors:** Karina Kapusta, Allyson McGowan, Santanu Banerjee, Jing Wang, Wojciech Kolodziejczyk, Jerzy Leszczynski

**Affiliations:** 1 Department of Chemistry and Physics, Tougaloo College, Tougaloo, MS 39174, USA; 2 Department of Chemistry, Physics and Atmospheric Sciences, Jackson State University, Jackson, MS 39217, USA

**Keywords:** SARS-CoV-2, mutations, molecular dynamics, protein–protein docking, AlphaFold, homology modeling, immune escape, benchmark

## Abstract

Even though COVID-19 is no longer the primary focus of the global scientific community, its high mutation rate (nearly 30 substitutions per year) poses a threat of a potential comeback. Effective vaccines have been developed and administered to the population, ending the pandemic. Nonetheless, reinfection by newly emerging subvariants, particularly the latest JN.1 strain, remains common. The rapid mutation of this virus demands a fast response from the scientific community in case of an emergency. While the immune escape of earlier variants was extensively investigated, one still needs a comprehensive understanding of how specific mutations, especially in the newest subvariants, influence the antigenic escape of the pathogen. Here, we tested comprehensive in silico approaches to identify methods for fast and accurate prediction of antibody neutralization by various mutants. As a benchmark, we modeled the complexes of the murine antibody 2B04, which neutralizes infection by preventing the SARS-CoV-2 spike glycoprotein’s association with angiotensin-converting enzyme (ACE2). Complexes with the wild-type, B.1.1.7 Alpha, and B.1.427/429 Epsilon SARS-CoV-2 variants were used as positive controls, while complexes with the B.1.351 Beta, P.1 Gamma, B.1.617.2 Delta, B.1.617.1 Kappa, BA.1 Omicron, and the newest JN.1 Omicron variants were used as decoys. Three essentially different algorithms were employed: forced placement based on a template, followed by two steps of extended molecular dynamics simulations; protein–protein docking utilizing PIPER (an FFT-based method extended for use with pairwise interaction potentials); and the AlphaFold 3.0 model for complex structure prediction. Homology modeling was used to assess the 3D structure of the newly emerged JN.1 Omicron subvariant, whose crystallographic structure is not yet available in the Protein Database. After a careful comparison of these three approaches, we were able to identify the pros and cons of each method. Protein–protein docking yielded two false-positive results, while manual placement reinforced by molecular dynamics produced one false positive and one false negative. In contrast, AlphaFold resulted in only one doubtful result and a higher overall accuracy-to-time ratio. The reasons for inaccuracies and potential pitfalls of various approaches are carefully explained. In addition to a comparative analysis of methods, some mechanisms of immune escape are elucidated herein. This provides a critical foundation for improving the predictive accuracy of vaccine efficacy against new viral subvariants, introducing accurate methodologies, and pinpointing potential challenges.

## 1. Introduction

**Figure 1:**
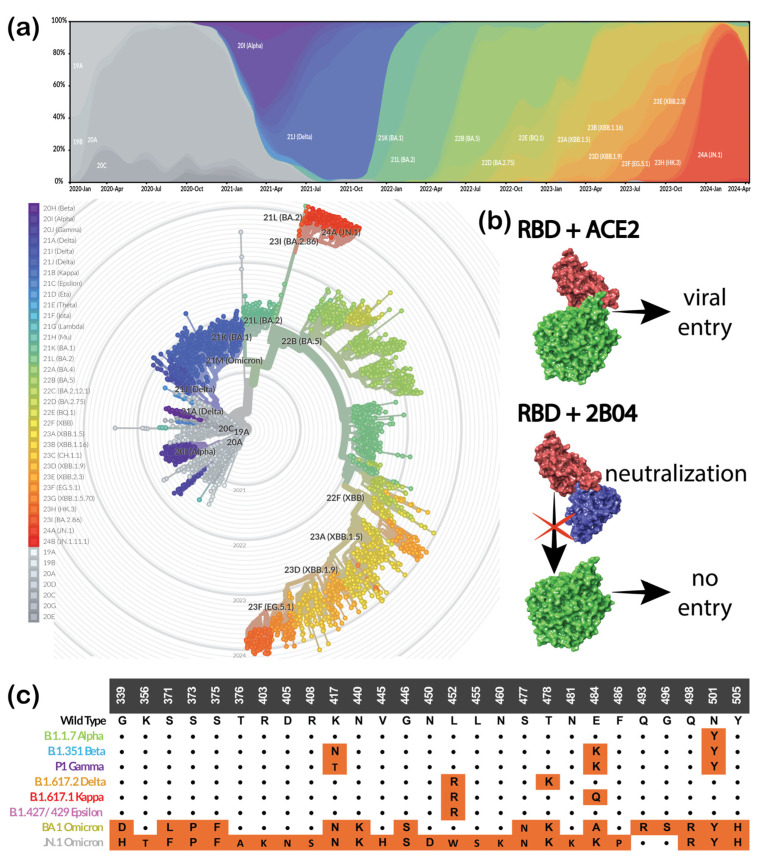
Mutations in SARS-CoV-2 receptor-binding domain: (**a**) mutation progression over time (Figures are adapted from Nextstrain.org, used under a CC-BY-4.0 license; https://nextstrain.org/ncov/gisaid/global/all-time (accessed on 1 May 2024)); (**b**) scheme of spike neutralization by 2B04 antibody; (**c**) list of mutations occurring in RBD of selected mutant structures.

Mutations in SARS-CoV-2, the virus that causes COVID-19, were a significant topic of research and concern throughout the pandemic. These mutations can alter the virus’s features, including its transmissibility, virulence, and interaction with the human immune system. Some mutations may increase the virus’s infectivity or transmissibility, while others might decrease them [1,2]. Viral evolution has led to the emergence of variants of concern, such as Delta and Omicron (since late November 2021) (Figure 1a), which raised concerns about possible immunity evasion and increased transmissibility [3]. Occurred mutations may impact the effectiveness of approved medications and vaccinations [4]. Thus, understanding their impact on viral properties is extremely important.

Significant progress has been made in understanding SARS-CoV-2 mutant immune escape using experimental methods. Classifications and structural analyses shed light on the immune responses against SARS-CoV-2 [5], providing a blueprint for the design of antibody cocktails as immunogens for vaccines. In [6], the spike glycoprotein’s N-terminal domain was identified as a promising target for therapeutic monoclonal antibodies (mAbs) against COVID-19. Specifically, the 4A8 mAb exhibited high neutralization potency against authentic and pseudotyped SARS-CoV-2 without binding to its receptor-binding domain (RBD). Eleven potent neutralizing antibodies against COVID-19 were identified in [7]. With a large number of experimental works concentrating on the Wild-Type (WT) RBD or selected mutants [5,6,7,8,9,10,11], some works provide a more comprehensive picture for the comparison of mutant immune escape [12,13,14,15,16]. The pseudovirus system makes deep mutational scanning of the complete SARS-CoV-2 spike possible. In [17], the genotype–phenotype-linked lentivirus pseudotyping method served as the basis for a mutational scanning platform to produce libraries of the Omicron BA.1 and Delta spikes. Nonetheless, the high cost of these assays makes evaluating a wide range of variants and strains experimentally challenging. This results in significant knowledge gaps that require further, more exhaustive, and more detailed investigation of the influence of mutations.

Computational methods allow for high-throughput analysis of big datasets, enabling researchers to systematically evaluate how mutations affect antibody binding and neutralization across various variants. Experimental studies alone may not capture the full spectrum of antigenic changes associated with SARS-CoV-2 mutants. Based on structure- and sequence-based analyses, computational approaches can predict the antigenic effects of mutations, offering insights into how mutations change the immune system’s detection of the virus. Molecular dynamic (MD) simulation and Molecular Mechanics/General Born Surface Area (MM/GBSA) have been shown to be promising tools to explore the effect of mutations on the binding affinity of monoclonal antibodies (mAbs) [18,19,20,21,22,23] and human angiotensin-converting enzyme (ACE2) [19,24,25,26,27] against the receptor-binding domain (RBD) region of the S protein. Various computational tools, such as AMBER (V 16, V 18, and V 20), GROMACS (V 2022.5), NAMD (V 2.13 and V 2.14), and Desmond (V 3.0), have been utilized. MD simulations are often enhanced by combining them with other approaches, such as density functional calculation [8] or FoldX 5.0, as used in [28] to predict escape mutations, focusing on the SARS-CoV-2 receptor-binding domain. Protein–protein docking is another widely employed approach for examining the RBD’s protein–protein interactions. This computational technique allows one to predict the binding pose and interactions between proteins with high precision. Various servers and tools are utilized to facilitate these studies. For instance, the ClusPro 2.0 server has been extensively used to model RBD–ACE2 interactions, providing valuable insights into their binding mechanisms [29,30]. Similarly, the HDOCK server supports a range of applications, including the analysis of RBD–ACE2 interactions [31], RBD interactions with amyloidogenic proteins [32], intraviral protein–protein interactions [33], and SARS-CoV-2 Orf7a/LFA-1 complexes [34]. The PatchDock and COVID-19 Docking Server were used for a drug discovery purpose, targeting RBD/ACE2 interactions using potential peptide-based drugs [35]. HawkDock was utilized in [36] to investigate the RBD–ACE2 interactions. As an alternative to physics-based methods, machine learning (ML) techniques have gained popularity and are widely implemented to study protein–protein interactions of SARS-CoV-2. These include an artificial intelligence-based framework, UniBind [37]; the Pipeline for the Extraction of Predicted Protein–Protein Interactions (PEPPI) [38]; various classifiers [39,40,41]; and language models [42], among others. The recently introduced AlphaFold 3 model, which is available for non-commercial use, demonstrates substantially high antibody–antigen prediction accuracy [43]. 

This work aims to compare and evaluate existing approaches and tools for the rapid and accurate assessment of antigen–antibody interactions, focusing on their potential influence on immune escape. As a benchmark model, we used experimental results from a study on SARS-CoV-2 neutralization by two murine antibodies targeting the RBD [13]. In this work, murine antibody 2B04 was shown to neutralize the spike glycoprotein RBD of specific variants by blocking its interactions with the host ACE2 receptor (Figure 1b). Errico et al. [13] performed structural comparisons that showed that 2B04 targets a distinct epitope in the RBD of the SARS-CoV-2 spike protein, overlapping significantly with the human 2–4 antibody. Both antibodies block ACE2 binding by targeting the RBM ridge, a key area for viral neutralization. However, the 2–4 antibody is known only to bind the “down” conformation of the RBD [44], while 2B04 bound both “up” and “down” conformations in [13]. In addition, this binding mechanism is shared with several other antibodies, namely CB6, B38, CC12.1, CC12.3, C105, COVA2-04, CV30, COVA2-39, REGN-10933, BD23, and P2B-2F6. This suggests that the computational insights gained from 2B04 can be applied to study and predict the functions of other neutralizing antibodies targeting the RBD–ACE2 binding interface, providing a template for broader antibody research. The wild-type (WT), Alpha, and Epsilon variants’ RBDs showed the highest affinity towards the antibody and were, thus, selected as positive controls. Meanwhile, the RBDs of Beta, Gamma, and Kappa exhibited immune escape and were chosen as decoys. Additionally, we investigated Delta and Omicron BA.1 and the newest Omicron JN.1, for which experimental data were unavailable at the time. The studied variants and mutations occurring in their RBDs are illustrated in Figure 1c.

In this study, we aimed to explore three fundamentally different approaches to assessing antigen–antibody interactions, each offering unique perspectives and advantages. These methods—(1) forced placement based on a template with further MD simulation utilizing Desmond, (2) protein–protein docking utilizing PIPER (both integrated into the Schrödinger Software Package), and (3) AlphaFold 3.0 antibody–antigen complex structure prediction—were chosen based on their proven accuracies in their respective categories of methods. Forced placement followed by MD simulation provides a comprehensive view of dynamic molecular interactions [45]. This approach is particularly advantageous for studying how mutations impact the flexibility and stability of protein complexes over time [46]. MD simulations allow us to observe real-time atomic movements, offering an in-depth understanding of how structural changes can influence binding affinity [47,48]. Unlike more static computational methods, MD simulations capture the conformational flexibility of proteins, which is crucial for identifying mutations that might lead to immune escape. This level of detail is fundamental when dealing with complex interactions and subtle structural changes. On the other hand, protein–protein docking offers a rapid and efficient way to predict binding poses and interactions between proteins [29,49,50]. This method is ideal for high-throughput analysis and initial screenings, as it quickly generates potential interaction models between the receptor-binding domain (RBD) and antibodies. While docking may not provide the same level of detail as MD simulations, it compensates with its speed and lower computational demands. Furthermore, docking does not require an accurate initial guess of the antigen geometry, which simplifies the process compared to MD simulations and allows for effective screening of numerous mutants. Finally, the recent advancements in AlphaFold, particularly with the introduction of AlphaFold 3.0, have significantly enhanced the accuracy of predicting antibody–antigen complexes. AlphaFold’s machine learning algorithm has demonstrated superior performance over traditional homology modeling in many cases [51,52]. AlphaFold’s advancements in the modeling of complex interactions represent a valuable tool for understanding how new mutations may affect antigen–antibody interactions, especially considering that it has not yet been tested for SARS-CoV-2 RBD/antibody complex prediction. 

Each method’s pros, cons, required application resources, and potential pitfalls are discussed further.

## 2. Materials and Methods

**Figure 2:**
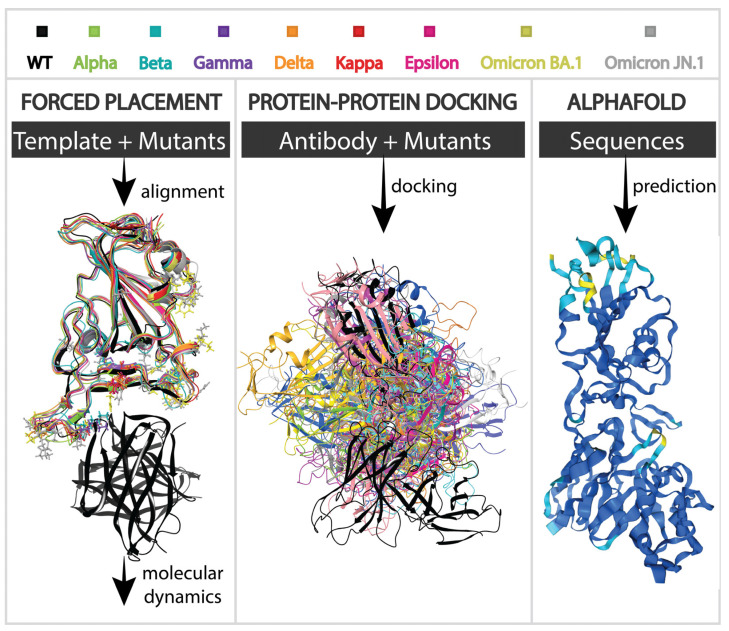
Schematic representation of approaches used in this study.

Each variant was assigned a color for illustration purposes—wild type (black), Alpha (green), Beta (cyan), Gamma (purple), Delta (orange), Epsilon (pink), Kappa (red), and Omicron BA.1 (yellow), and Omicron JN.1 (grey)— which are maintained throughout this manuscript (Figure 2). The structures of a SARS-CoV-2 wild type complexed with murine antibody 2B04 (PDB ID: 7K9I) and RBDs of Alpha (B.1.1.7), Beta (B.1.351), Gamma (B.1.128), Delta (B.1.617.2), Epsilon (B.1.429), Kappa (B.1.617.1), and Omicron BA.1 (B.1.1.529) were retrieved from the Protein Databank (https://www.rcsb.org/ (accessed on 1 January 2024)) with PDB IDs 7EDJ, 7LYK, 7NXC, 7W9I, 7N8H, 7V87, and 7T9L, respectively. Each structure was prepared using the Schrödinger Software Package (Schrödinger Release 2024-1: Schrödinger, LLC, New York, NY, USA, 2024). SARS-CoV-2 mutant spikes were trimmed to contain only their RBDs. Chain A was used for each variant, except for the Gamma and Delta variants, where chain B and chain E were used as RBDs, respectively. Every target protein was prepared using Protein Preparation Wizard. The bond orders were assigned, and all hydrogen atoms were replaced. Protonation states were generated using Epik for a pH of 7.4 ± 0.0 (physiological pH), the hydrogen bond network was optimized, and restrained minimization of all atoms was carried out using OPLS4e force fields [53]. The importance of glycosylation in studying the RBD interactions has been numerously revealed. In [54], it was shown that deglycosylation reduces the interaction strength between SARS-CoV-2 and SARS-CoV RBDs and ACE2 protein. Nonetheless, being reduced overall, the dissociation constants maintained similar trends, with the SARS-CoV-2 RBD binding stronger than that of its predecessor. Moreover, as shown in [13], the glycans on the RBD do not contribute to the binding of 2B04. Thus, our work does not involve the use of glycans to simplify mutant comparison models. The hydrophobic/hydrophilic surface was calculated for the prepared WT RBD/2B04 complex.

**Figure 3:**
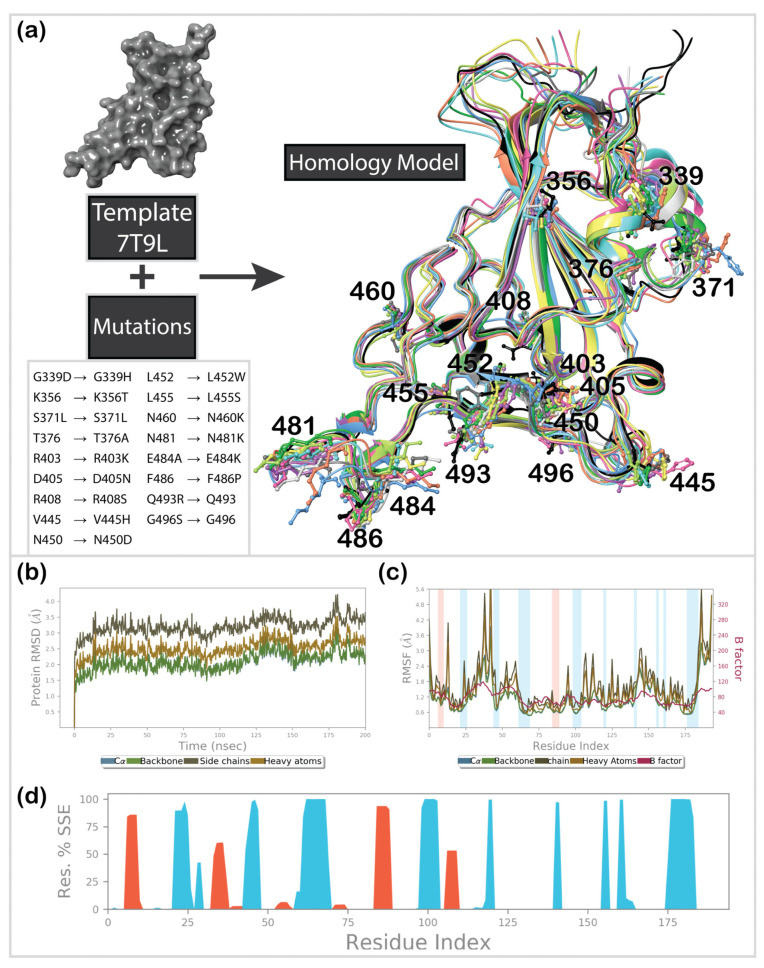
MD simulation of the Omicron JN.1 RBD’s homology model: (**a**) scheme of a general homology modeling approach and superposition of the 10 most populated clusters (colored ribbons) after 200 ns MD simulation and reference Omicron BA.1 structured (black ribbons) with labeled mutated residues; (**b**) RMSD plots; (**c**) RMSF plots; (**d**) secondary protein structure.

The 3D structure of the newly emerged JN.1 subvariant of Omicron SARS-CoV-2 RBD was unavailable in the Protein Databank at the time of the study. Thus, homology modeling was used to recreate it. We used the GISAID Nextstrain database (https://nextstrain.org/ncov/gisaid/global/all-time (accessed on 1 January 2024)) to retrieve information regarding the mutations that occurred in RBD. For the majority of the reported sequences, these mutations were G339H, K356T, S371F, S373P, S375F, T376A, R403K, D405N, R408S, K417N, N440K, V445H, G446S, N450D, L452W, L455S, N460K, S477N, T478K, N481K, E484K, F486P, Q498R, N501Y, and Y505H (Figure 1c). A model was created using Schrödinger’s Homology Modeling module, with the original Omicron BA.1 (PDB ID: 7T9L) used as a template (Figure 3a). Secondary structure prediction was carried out using the ClustalW alignment method, which works best for structures with high sequential similarity [55]. The model was aligned and further built using a knowledge-based method. A molecular dynamics simulation was performed to equilibrate the structure. The system was prepared by adding six chlorine anions for neutralization and filled out using the SPC water solvation model in the orthorhombic minimized periodic box. The OPLS4e force field was used with Desmond’s [56] default eight-stage relaxation protocol, which preceded the actual run. The model was subjected to 200 ns molecular dynamics simulation for structure refinement, as suggested in our previous work [45]. RMSD and RMSF plots were built to validate the quality of the created model. The most populated cluster generated by the Desmond trajectory clustering method was used for further calculations.

For the forced alignment method, prepared mutants’ RBDs were aligned based on a template WT RBD/2B04 complex (PDB ID: 7K9I), as shown in Figure 1b. The WT RBD of this complex was substituted by mutant complexes to create the initial structures of Alpha, Beta, Gamma, Delta, Epsilon, Kappa, Omicron BA.1, and Omicron JN.1 complexes. Complexes were further subjected to short 100 ns MD simulations utilizing the Desmond module with the same settings as described for equilibration of the homology model, except the solvation box size was set to be buffered at 10 × 10 × 10 Å for all complexes. The trajectory frame clustering method was used to determine the most populated structures throughout the trajectory. These structures (equilibrated complexes) were placed in a 20 × 20 × 20 Å orthorhombic periodic buffer box and solvated with SPC water molecules. The trajectory clustering method was used again to retrieve the final structures, which were analyzed using the simulation interaction diagram and protein interaction analysis. For each complex, the sum of formed hydrogen bonds (HBs), salt bridges, π-stacking, vdW clashes, surface complementarity scores, and buried-solvent accessible surface area scores (SASAs) was calculated. Energies were calculated using Prime.

Protein–protein docking was carried out using PIPER [57]. The antibody from the prepared WT RBD/2B04 complex was extracted, and all mutants’ RBDs were docked into it as antigens with a default of 70,000 antigen rotations to probe and a maximum of 30 poses being refined and reported as outputs. This approach used five different settings: default all-structure docking, docking with masked non-CDRs (complementarity-determining regions), and docking with masked non-CDRs and set constraints. We analyzed the geometry of the prepared WT RBD/2B04 complex to set the specific constraints. F486 was identified as an essential residue of the RBD that fits into a tight hydrophobic pocket of the antibody near its residue, Y34. The distance between these residues was set as a first constraint to vary between 2 and 10 Å for the first case of docking with masked non-CDRs and set constraints. A second constraint was added to the second docking case with masked non-CDRs and set constraints. In addition to the F486/Y34 pair, we fixed the distance between the antigen’s G446 and the antibody’s Q1 residues to vary between 2 and 10 Å. Finally, the last setting used the same two constraints but with a more significant variation of distances (between 2 and 20 Å) to allow more flexibility. All 30 poses for each complex produced by protein–protein docking were analyzed by their PIPER pose energy, PIPER pose score, and RMSD aligned on the prepared WT RBD/2B04 complex. The specific score was calculated using Formula (1) to fit experimental data.

(1) $$PPI\;Score=\frac{pose\;number}{number\;of\;poses}\times RMSD$$

For poses with the smallest RMSD, protein interaction analysis was performed.

Finally, the third method used in this work was complex structure prediction utilizing the AlphaFold Server (https://alphafoldserver.com/ (accessed on 20 June 2024)). [35] For this approach, the sequences of the antibody and all RBDs were retrieved in FASTA format and used as inputs for the complexes’ structure prediction. The following seeds were used for the WT, Alpha, Beta, Gamma, Delta, Kappa, Epsilon, Omicron BA.1, and Omicron JN.1 RBD/antibody complexes: 1505197086, 759516498, 1264705315, 2105578610, 1939194179, 91611597, 1799657852, 476548122, and 2128069589, respectively. The predicted template modeling (pTM) and the interface-predicted template modeling (ipTM) scores, which measure the accuracy of the structure, were retrieved from this web server. Additional scoring was performed using the ranking score, which incorporates pTM; ipTM; the fraction of the prediction structure that is disordered (disorder), as measured by accessible surface area; and the relative number of clashing atoms (has_clash) in a single number expressed by the following equation:

0.8 × *ipTM* + 0.2 × *pTM* + 0.5 × *disorder* − 100 × *has_clash* (2)

The obtained structures were further exported and analyzed in the Maestro interface to calculate their RMSDs aligned on the prepared WT RBD/2B04 complex.

## 3. Results

### 3.1. Homology Modeling

Structure-based computational approaches have certain pitfalls, as they require the availability of a 3D structure. With the extensive mutation rate of SARS-CoV-2, only the most concerning variants and mutant structures have been analyzed and deposited in the Protein Databank. This creates a knowledge gap and limits structure-based investigations. One often knows only the sequences of newly emerging variants for a significant amount of time. In such cases, homology modeling is a valuable tool for structure prediction. This study used the Omicron BA.1 RBD template structure (PDB ID: 7T9L) to create a model of the newly emerged Omicron JN.1 mutant (Figure 3a). As shown in our previous works [45,58], molecular dynamics (MD) simulation aids in further equilibration of the built model. Analysis of the MD trajectory provides decent validation for a model built using this approach. The root-mean-square deviation (RMSD) was analyzed for Cα, the backbone, side chains, and all heavy atoms (Figure 3b), indicating no significant movement throughout the 200 ns simulation time. The variance of deviations within the trajectory did not exceed 1.5 Å, suggesting high structural stability. Similarly, the root-mean-square fluctuation (RMSF) plot (Figure 3c) did not show significant fluctuations in the backbone and Cα. Slightly higher fluctuations were noticed for side chains and heavy atoms in loop areas, specifically between residue numbers 368–383, located far from the binding interface of the spike’s RBD. Finally, the secondary structure was mainly maintained throughout the trajectory, as shown in Figure 3d. After validation of the developed model, it was safe to assume that it could be used for further investigations.

### 3.2. Forced Placement and MD Simulations

The first method tested here was forced placement based on an existing template of wild-type SARS-CoV-2 complexed with murine antibody 2B04 (PDB ID: 7K9I). To create preliminary models of the complexes, the wild-type RBD was substituted with that of each mutant. Unoptimized structures would not provide any legitimate information on complex stability. Thus, the preliminary complexes were subjected to a 100 ns molecular dynamics simulation to refine protein–protein interactions in a realistic environment.

**Figure 4:**
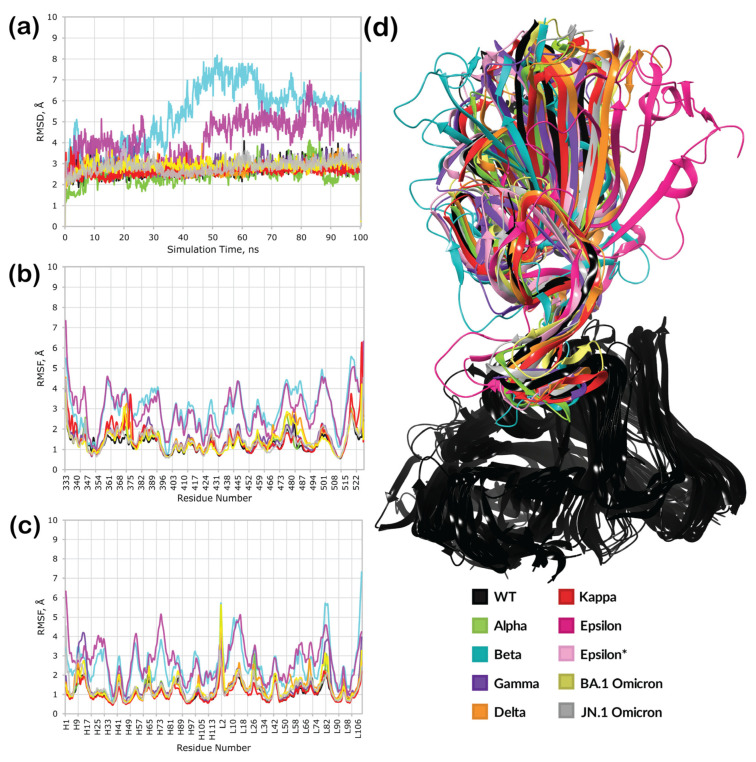
Results of the preliminary 100 ns molecular dynamics simulations: (**a**) RMSD plots; (**b**) RBD RMSF plot; (**c**) antibody RMSF plot (H and L stand for antibody subunits); (**d**) superposition of the most populated clusters from the molecular dynamics trajectory (Epsilon* is the fifth most populated cluster with the lowest RMSD). Average GPU time: 6H 28′ 54″; average total rate per step: 375.36 ns/day; Linux-x86_64. Resources: Z8G4 2.4 GHz Intel Xeon Silver 4214R 12-Core 64GB 2933 MHz DDR4 ECC Registered RAM NVIDIA Quadro RTX A5000 1TB.

This trajectory’s RMSD and RMSF plots (Figure 4a,b) showed high stability for all variants except Beta and Epsilon. While it was expected that the Beta RBD would have little to no binding affinity towards the 2B04 antibody, the low stability of the Epsilon variant was unexpected, given its reported high neutralizing power. Interestingly, not only did the RBDs of these variants exhibit high fluctuations, but the antibody itself became less stable, as seen in its RMSF (Figure 4c).

**Figure 5:**
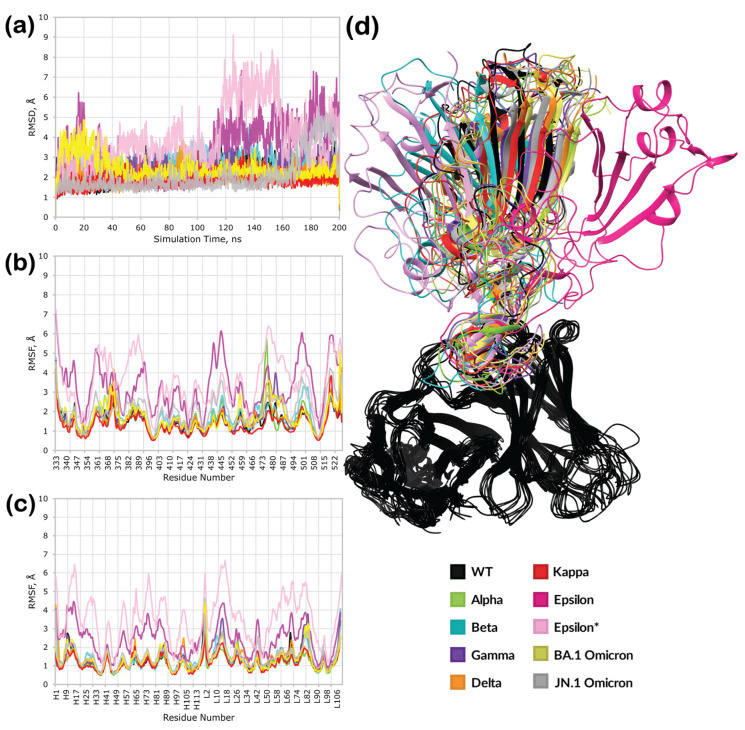
Results of the 200 ns molecular dynamics simulations: (**a**) RMSD plots; (**b**) RBD RMSF plot; (**c**) antibody RMSF plot (H and L stands for antibody subunits); (**d**) superposition of the most populated clusters from the molecular dynamics trajectory (Epsilon* is a 200 ns simulation with the initial structure derived from the fifth most populated cluster of the 100 ns preliminary MD simulation). Average GPU time: 45H 19′ 12″; average total rate per step: 106.40 ns/day; Linux-x86_64, Resources: Z8G4 2.4 GHz Intel Xeon Silver 4214R 12-Core 64GB 2933 MHz DDR4 ECC Registered RAM NVIDIA Quadro RTX A5000 1TB.

After clustering the obtained trajectories, we chose the most populated cluster for each variant (Figure 4d, Supporting Table S1). However, to further elucidate the unexpected trajectory of the Epsilon complex, an additional structure was extracted for further investigation. We attempted to select the complex that most closely resembled the template wild-type RBD complex. For Epsilon, this was the fifth most populated cluster. Each equilibrated complex was subjected to an additional 200 ns MD simulation, this time in a twice as sizeable periodic box (to provide more space for a potential dissociation of a complex). The results of this run are illustrated in Figure 5.

Interestingly, the Beta RBD–antibody complex showed a stable trajectory and low overall fluctuations this time (Figure 5a–c), indicating that the previous 100 ns run followed by clustering helped equilibrate the complex. Similarly, complexes of other mutants were stable, with only a slight change in RMSD for the JN.1 complex after 160 ns of simulation (Figure 5a). However, the second run did not resolve the instability in the case of the Epsilon complex. Structures from both the first and fifth most populated clusters from the equilibration run showed significant deviations and fluctuations for both the RBD and the antibody. Clustering revealed that both trajectories resulted in complexes with low similarity to the template structure (Supporting Table S2, Figure S1).

**Figure 6:**
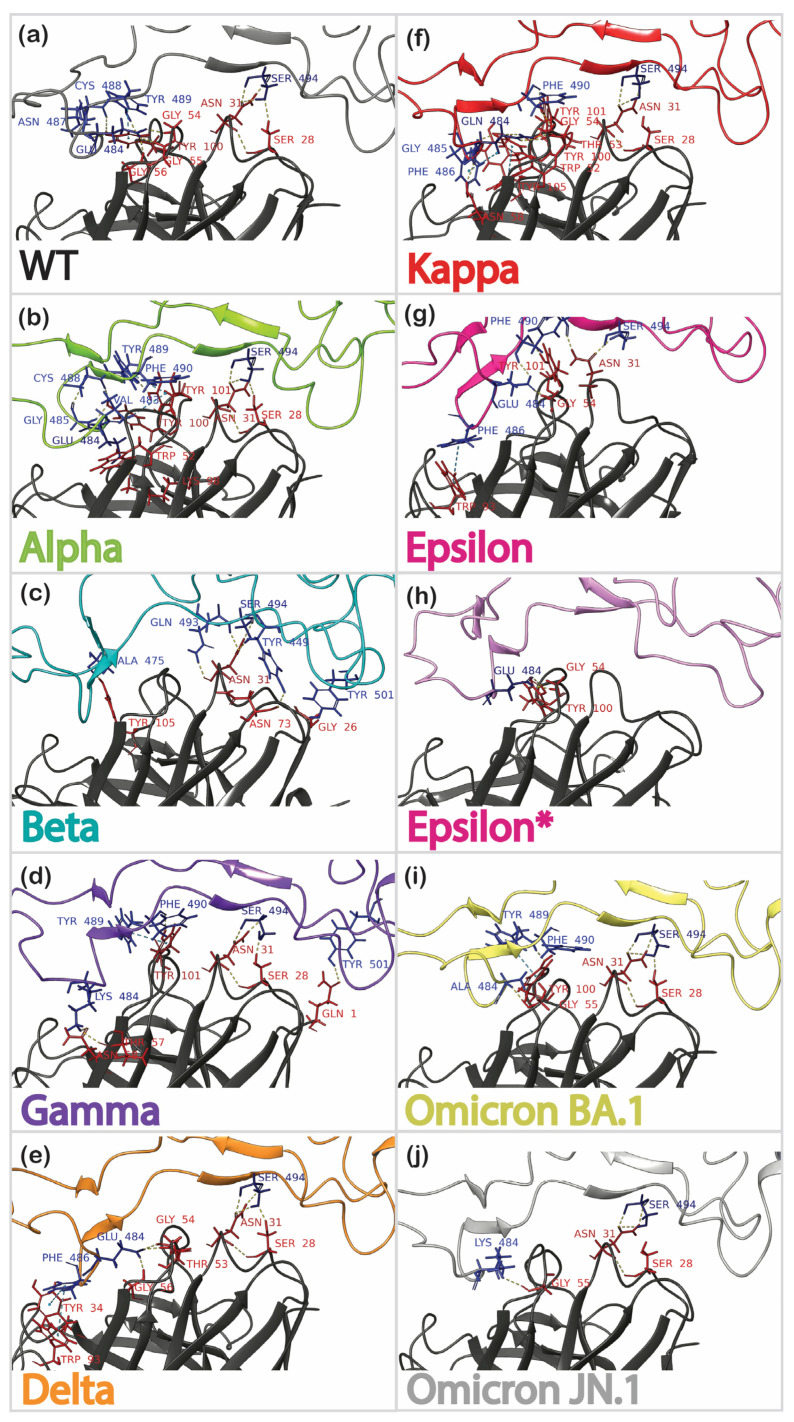
Protein–protein interactions for the most populated clusters of a 200 ns MD simulation of the RBD (residues colored in blue) complexed with the 2B04 (residues colored in red) antibody: (**a**) WT; (**b**) Alpha; (**c**) Beta; (**d**) Gamma; (**e**) Delta; (**f**) Kappa; (**g**) Epsilon; (**h**) Epsilon* (with the initial structure derived from the fifth most populated cluster of the 100 ns preliminary MD simulation); (**i**) Omicron BA.1; (**j**) Omicron JN.1.

Clustered complexes were extensively analyzed through protein interaction analysis (Supporting Tables S3–S12), as summarized in Table 1. As expected, the highest number of interactions was observed for the wild-type complex, forming ten hydrogen bonds and π–π stacking between Y489 of the spike and Y100 of the antibody (Figure 6a). Critical residues for hydrogen bonding included E484, N487, C488, and S494. Of these residues, only E484 was mutable, while the other four were conserved throughout all mutants studied here. Strong interactions were also observed for the Alpha complex, yielding eight hydrogen bonds, one salt bridge, one instance of π–π stacking, and one van der Waals (vdW) clash. The N487 residue was not crucial for the formation of the complex, while the other residues that participated in the formation of the wild-type complex were also present (Figure 6b). The Beta, Gamma, and Delta complexes had fewer interactions than the first two complexes (Figure 6c–e). However, the difference was not as significant as expected based on experimental data. An unexpectedly high number of interactions was predicted for the Kappa variant’s complex, which formed eight hydrogen bonds, one instance of π–π stacking, and one vdW clash (Figure 6f), similar to the Alpha variant’s complex. Overall, the fewest interactions were observed in the trajectories of complexes of the Epsilon (Figure 6g,h) and Omicron mutants (Figure 6i,j) with antibodies. While these results were expected for the Omicron spikes, the Epsilon results were predicted to be false negatives, considering the available experimental data.

Interestingly, all complexes except for the first Epsilon complex (Figure 6g) showed the S494 residue of RBD being conserved to form hydrogen bonds with the N31 residue of the antibody.

### 3.3. Protein–Protein Docking

The next tested approach was protein–protein docking, which was implemented in Schrödinger’s PIPER module. Table 2 summarizes the results of testing five different settings for WT antibody/antigen docking. The first setting (default algorithm) yielded a relatively accurate prediction, with the most populated pose having an RMSD of 7.646 Å compared to the prepared crystallographic complex. The remaining poses had RMSD values ranging from 10 Å to 40 Å. Masking the non-CDR region (second setting) improved the overall accuracy, resulting in RMSD variations not exceeding 34 Å. However, the most populated cluster wasn’t the one with the lowest RMSD in this case. Pose 4 yielded the most accurate result, with an RMSD of 3.63 Å compared to the crystallographic structure. 

We expected the addition of constraints to reduce variations and improve accuracy. A careful analysis of the geometry of the prepared WT RBD/2B04 complex was required to set specific constraints. F486 was identified as an essential residue of the RBD that fits into a tight hydrophobic pocket of the antibody near its Y34 residue. The distance between these residues was chosen as the first constraint and set to vary between 2 and 10 Å for the third setting. In the case of the fourth setting, an additional constraint was added. One more pair of residues was restrained, with the distance between the antigen’s G446 and the antibody’s Q1 residues fixed to vary between 2 and 10 Å. For the fifth and the last setting, both constraints were used, with the distance increased to allow more flexibility, ranging from 2 to 20 Å. Interestingly, restrained docking did not improve accuracy; the lowest RMSD value of 6.091 Å was measured when two residues were restrained within 20 Å. This value was still more significant than the one for the masked non-CDR without any constraints. 

**Figure 7:**
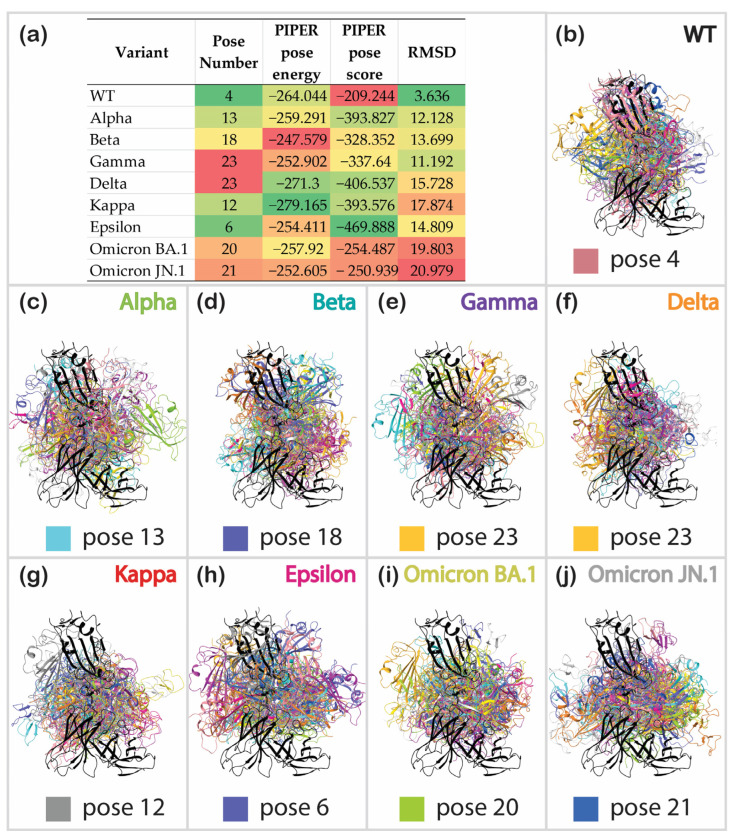
Results of protein–protein docking: (**a**) pose numbers of the docked structures with the corresponding lowest RMSD value, PIPER pose energy and score, and RMSD for each complex; superposition of all 30 docked poses on the reference structure (colored in black) for (**b**) WT; (**c**) Alpha; (**d**) Beta; (**e**) Gamma; (**f**) Delta; (**g**) Kappa; (**h**) Epsilon; (**i**) Omicron BA.1; (**j**) Omicron JN.1 (the color of the pose with the lowest RMSD and its order are illustrated).

Based on the benchmark results (Table 2), protein–protein docking with the masked non-CDR and no constraints was used for all further calculations. Similar to the results for the WT RBD/antibody complex, protein–protein docking for most other variants produced at least one pose closely resembling the template. However, it was never the most populated pose. The pose numbers of the lowest RMSD structure, the PIPER pose energy and score, and the pose RMSD were collected and analyzed (Figure 7a, Supporting Table S13). The WT complex exhibited the lowest RMSD among mutants, followed by Gamma, Alpha, Beta, Epsilon, Delta, Kappa, Omicron BA.1, and Omicron JN.1, which has highest RMSD. Although Gamma and Beta had significantly lower RMSD values, the poses exhibiting these RMSDs were not the most favorable, ranked 18th and 23rd, respectively. It must be noted that the docking energy and score can rarely be used for scoring. In our study, these values did not correlate with the experimental data, and the only accurate method to rank complexes was comparison of their RMSD values for specific poses. Figure 7b–j illustrate the superposition of all 30 poses for each variant complex, with the lowest RMSD pose marked at the bottom of each picture. Despite the high variance of poses for all complexes, the docked poses for the positive controls, WT (Figure 7b), Alpha (Figure 7c), and Epsilon (Figure 7h) often overlapped with the reference structure. In contrast, the docked poses of decoys, such as the Kappa (Figure 7g), as well as Delta (Figure 7f), Omicron BA.1 (Figure 7i), and Omicron JN.1 (Figure 7j) complexes, were significantly different from the reference. However, the results for Beta and Gamma were not entirely conclusive, as they showed relatively low RMSDs. Protein–protein interactions for the poses of each complex were analyzed, as highlighted in Figure 7a. We hoped to find that, disregarding the low RMSDs, Beta and Gamma RBD/antibody complexes would show fewer interactions. However, the data (Table 3) brought even more inconclusiveness, showing WT and Kappa complexes as the most favorable based on the number of formed interactions. None of these values correlate significantly with experimentally known activities, leading us to rely on RMSD scoring as the most convenient option. 

### 3.4. Sequence-Based Prediction with AlphaFold

**Figure 8:**
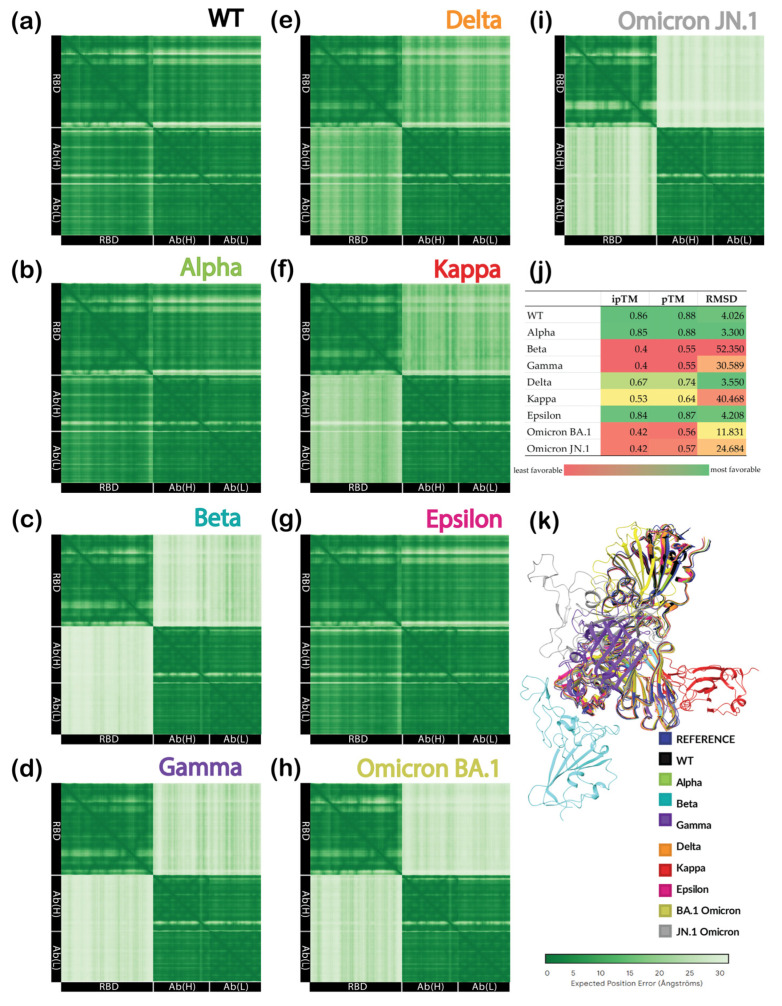
Results of AlphaFold prediction: predicted aligned error (PAE) matrix (darker is more confident) for complexes of (**a**) WT; (**b**) Alpha; (**c**) Beta; (**d**) Gamma; (**e**) Delta; (**f**) Kappa; (**g**) Epsilon; (**h**) Omicron BA.1; (**i**) Omicron JN.1; (**j**) prediction scores and RMSDs compared to the reference structure; (**k**) superposition of complex predictions based on the reference structure (colored in blue).

Finally, we tested one more approach for scoring the stability of protein–protein complexes. Since protein–protein docking scores did not correlate with experimental data and RMSD emerged as the sole determinant of complex stability, we applied AlphaFold to predict the complex structure. The antibody and all RBD sequences were submitted to the AlphaFold Server (Supporting Table S14). All predictions were completed within 2 min, except for those for the Kappa and Omicron BA.1 complexes with an antibody, which were done within 1 min. Position error maps are collected in Figure 8a–i. The highest prediction confidence was observed for the WT, Alpha, and Epsilon RBD complexes with antibodies. These models showed the highest values of ranking score, pTM, and ipTM, along with the lowest RMSDs (Figure 8j). Conversely, the Beta, Gamma, and Omicron’s subvariant RBDs exhibited the lowest structural confidence levels and ranking scores, with RMSD values of 11 Å and above. The Kappa RBD complex showed the second highest deviation from the reference structure with average confidence scores. Significantly, all findings correlated well with experimental data, yielding the highest predictive ability. The superpositions of all complexes aligned on the reference WT/2B04 structure are illustrated in Figure 8k.

## 4. Discussion

After testing these three methods, we identified the pros and cons of each. We retrieved experimental data for immune escape from [13] to evaluate more accurate correlations between the experimental data and predictions. The half-efficacy concentration (EC_50_) values of neutralization for the WT, Alpha, Beta, Gamma, Kappa, and Epsilon viral strains by 2B04 antibody (ng/mL) were converted into pEC_50_ (−log(EC_50_)) and calculated as 0.2218, 0.7959, −4.0000, −4.0000, −4.0000, and −0.0500, respectively.

**Figure 9:**
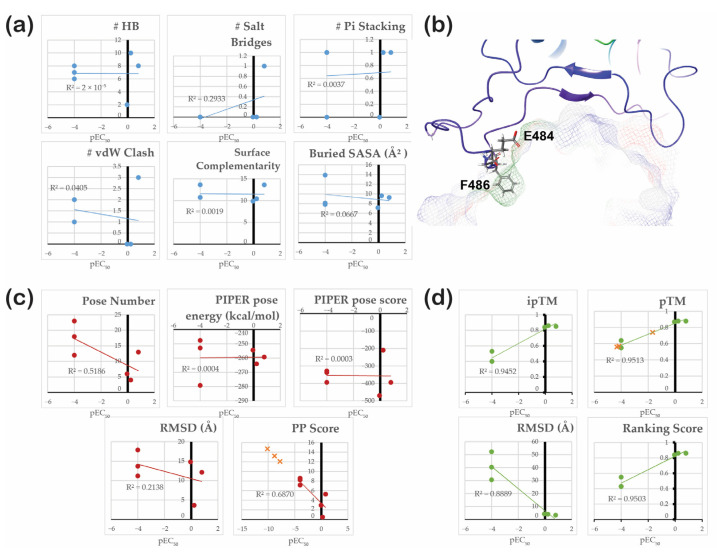
Correlation between predicted parameters with experimental data and general discussion: (**a**) results of a forced placement approach; (**b**) hydrophobic/hydrophilic surface of an antibody and the role of critical residues E484 and F486; (**c**) results of protein–protein docking; (**d**) results of AlphFold prediction.

For the forced placement, we did not notice any decent correlations with the experimental data, as shown in Figure 9a. More/less accurate predictions could be made by categorizing variants’ RBDs as those neutralized by antibodies and those escaping antibody activity, followed by further qualitative assessment. Summing up the total number of interactions (Table 1) might suggest that those with over ten interactions are neutralized, while those with fewer than ten escape. However, this categorization showed a false-negative result for the Epsilon RBD/antibody complex and a false-positive result for the Kappa RBD/antibody complex. A molecular mechanics (MM/GBSA) approach can be implemented to improve prediction accuracy to evaluate the binding free energy of complexes. However, as this method requires accurate initial structures, it would improve the results only slightly, considering that the most populated cluster of Epsilon is not fully bound and that the Kappa cluster indicates a large number of interactions.

Furthermore, we attempted to explain the reason behind the unsuccessfully modeled complexes. In the case of the Kappa variant of SARS-CoV-2, only two mutations occurred within the RBD (L452R and E484Q). The E484 residue mutation is well studied and has attracted interest due to its correlation with the variants of concern at the time, such as the Beta and Gamma variants. This mutation has also been observed for various Omicron subvariants. The substitution of a negatively charged glutamic acid (E) with a positively charged lysine (E484K) (as in the case of Beta, Gamma, and Omicron JN.1 in this work) resulted in decreased binding affinities [59,60,61,62]. Similarly, the effect of this residue’s substitution with neutral alanine (E484A) or glutamine (E484Q) resulted in enhanced immune escape, with E484A being slightly better than E484Q [63]. The effect of the E484K (Q, A) mutation on the neutralization of RBDs by antibodies was explained mainly through electrostatic and hydrophobic interactions, as well as hydrogen bonding at the binding interface of the RBD and different neutralizing antibodies [18]. 

Here, we propose a more in-depth explanation of the role of this mutation. When analyzing the hydrophobic/hydrophilic surface of a 2B04 antibody (Figure 9b), the tight hydrophobic pocket was identified in place of the interactions with the RBD’s F486 residue. It is located near E484, a negatively charged residue that forms hydrogen bonds and salt bridges with the antibody residues. Its substitution with the positively charged lysine (K) hydrophobic alanine (A) results in the repulsion of the whole region; thus, F486 cannot enter the hydrophobic pocket of the antibody. The formation of a salt bridge becomes impossible when substituting with glutamine (Q), which has a polar acidic side chain. Nonetheless, molecular dynamics simulation still shows the formation of strong hydrogen bonds due to a high level of structural similarity of glutamic acid and glutamine (as was shown in this work and the work of Gupta et al. [18]). When a forced placement method was used, the E484Q mutation could only slightly decrease interaction power. Still, this mutation did not cause a strong repulsion (as in the case of the Beta, Gamma, and Omicron subvariants’ complexes). Thus, having the F486 residue forcedly placed inside the antibody’s hydrophobic pocket provided an additional attraction to hold the complex together, resulting in a false-positive result for Kappa. This hypothesis is also supported by other research on the influence of mutations on antibody efficiency, which indicated E484A, E484G, E484K, and F486S as the most important mutations for the reduced neutralization of the VSV-SARS-CoV-2 mutants by the 2B04 antibody [15].

Contrary to MD simulation, protein docking required much less time and computing power. Nonetheless, the pitfall of this method was low structure similarity for the highest-ranking pose. The manual assessment of all produced poses (or simple RMSD calculation) was required to identify the most accurate and realistic binding pose. Considering that the 2B04 antibody targets the RBD–ACE2 interface, if the resulting binding modes show an antibody interacting with non-RBM regions, these complexes can be roughly assumed to possess immune escape potential. In such cases, the RMSD value for binding poses, using the crystallographic structure of the wild-type RBD-2B04 complex (PDB ID: 7K9I) as a reference, can serve as a rough indicator of a variant’s immune escape. This approach is particularly useful when crystallographic data for other variants are unavailable. The PIPER pose energy and the PIPER pose score of neither the lowest- RMSD nor highest-ranking poses showed any correlations with the experimental values (Figure 9c). Slightly better correlations were noted for the RMSD (R^2^ = 0.2138) and the pose number (R^2^ = 0.5186). The low correlation of the RMSD could be explained by the example of the Gamma RBD/antibody complex. It showed a relatively low RMSD of 11.192 Å, but this RMSD belonged to the pose ranked 23rd by population (Figure 7), while prior poses had significantly higher RMSD values (Supporting Table S12). The less populated the pose is, the less probable it is to exist. That gave us the idea to using Protein–Protein Interaction (PPI) score calculated by Equation (1) provided in the Methods section. This score allowed us to rank the strength of the RBD/antibody complexes quickly yet relatively accurately (R^2^ = 0.6870) without requiring time-consuming averaging of all produced poses’ RMSDs across their populations. This method predicts Delta and Omicron subvariants’ complexes (plotted as orange crosses in Figure 9c) to have a significantly greater immune escape capability than other variants. This prediction sounds legitimate, as the variants mentioned above are known for their strong immune evasion capabilities relative to other antibodies. With respect to the PPI score, except for the Alpha complex and the WT complex, which were predicted to be too low, this approach did not show any clear outliers. Nonetheless, considering the complexes mentioned above as outliers and the low R^2^ value of this relationship, we concluded that this method is better for categorizing. Complexes with a score above six do not interact strongly with the antibodies, while those with a score below six can be neutralized by the antibodies.

Finally, the prediction of complex structures with AlphaFold showed surprisingly high correlations with the experimental data (Figure 9d), with R^2^ values for ipTM, pTM, ranking score, and RMSD equal to 0.9452, 0.9513, 0.9503, and 0.8889, respectively. The RMSD values for the best-scoring structure predicted by AlphaFold were superior to those calculated by PIPER protein–protein docking, with only one data point raising concern. The AlphaFold server predicted a very low RMSD for the complex of the Delta variant (Figure 8j), which was suspected to escape antibody neutralization. Nonetheless, the confidence in this prediction was rather low, as illustrated in Figure 8e. At first, the unexpected correlation with probability-related scores, such as ipTM and pTM, was suspicious. We hypothesized that it could be related to the fact that available databases used by the AlphaFold Server for model training include larger amounts of data for the earlier variants, which many antibodies could easily neutralize. In contrast, the data for the newer variants exhibiting immune escape were not as robust. This could cause a fake correlation based on the pure availability of data rather than the influence of mutations. To test this hypothesis, the non-existent sequence of the SARS-CoV-2 mutant’s RBD was constructed such that it would exhibit five different mutations, all in the area outside of the RBD/antibody binding interface. These random mutations included V362I, A363L, V367A, L368I, and A372L. The modeled structure had low confidence in the areas near mutations. However, it maintained very high/high confidence at the binding interface with ipTM = 0.84 and pTM = 0.87, the same as the Epsilon complex and only slightly inferior to the Alpha complex. The high confidence in predicting the non-existent variant, which did not feature mutations that could facilitate immune escape, disproved the previous hypothesis and increased our trust in this method. As the pTM values illustrated the highest correlation with the experimental data, they were used to predict the neutralization efficiency of the antibodies against the Delta variant and Omicron BA.1 and JN.1 subvariants, as plotted as orange crosses in Figure 9d. This prediction suggest that the Omicron subvariants exhibited the highest levels immune evasion, while Delta RBD was predicted to have achieve a medium level of immune escape. Among the methods tested here, structure prediction with AlphaFold was shown to be the least time- and resource-consuming and, simultaneously, the most accurate for scoring the neutralizing power of antibodies.

**Figure 10:**
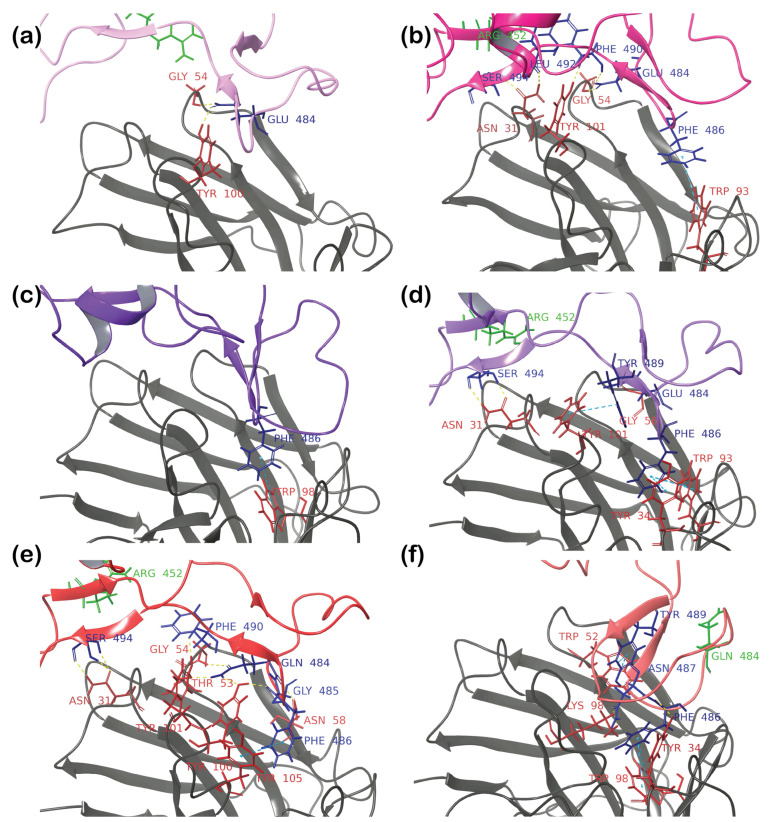
Protein–protein interactions predicted by different approaches: (**a**) Epsilon-2B04 complex as a result of a forced placement (model 1); (**b**) Epsilon-2B04 complex as a result of a forced placement (model 2); (**c**) Epsilon-2B04 complex as a result a protein–protein docking; (**d**) Epsilon-2B04 complex as a result of an AlphaFold prediction; (**e**) Kappa-2B04 complex as a result of a forced placement; (**f**) Kappa-2B04 complex as the result of protein–protein docking.

The analysis of all methods provided greater insight into the factors contributing to false-positive and false-negative predictions, with a particular focus on antibody complexes with Epsilon and Kappa RBDs. The first model generated via forced placement (Figure 10a) identified a single RBD residue, i.e., E484, interacting with the antibody and forming two hydrogen bonds with residues G54 and Y100. The bond with G54 appeared reliable, as it was consistent across both the second forced placement model and the AlphaFold-predicted complex. Molecular dynamics (MD) simulations can be sensitive to the initial structural guess, potentially trapping the system in a local energy minimum if the starting geometry is inaccurate. This issue can be addressed by initiating simulations from a different starting structure, as was done for the second model of the Epsilon RBD-2B04 complex built using the forced placement (Figure 10b). Protein–protein docking also showed sensitivity to the initial antibody structure. Similar to the first model of forced placement, it revealed only one interaction between the antigen and antibody—a π–π interaction between F486 and W98 (Figure 10c). Despite the antigen’s flexibility, the antibody structure remained rigid during docking, mirroring the geometry used in the forced placement method. This rigidity likely contributed to inaccuracies. Thus, assuming that a wild-type RBD–antibody complex is a suitable template for all variants is not always appropriate and can introduce significant errors, particularly in PPD when a rigid antibody structure is used.

These limitations did not restrict the predictive AlphaFold approach, rapidly producing a complex similar to the refined forced placement (model 2) (Figure 10b). Both approaches identified critical interactions, including an H bond between the RBD’s S494 and the antibody’s N31, π–π interactions involving the RBD’s F486 and the antibody’s W93, and the importance of RBD residue E484 and antibody residue Y101 (with slightly different interactions observed in the two methods). 

For the Kappa RBD, interactions with antibodies were overestimated using both forced placement and PPD (Figure 10e,f). As mentioned earlier, the E484Q mutation introduced significant repulsion, preventing F486 from fitting into the antibody’s hydrophobic pocket. In the PPD model, F486 incorrectly entered this pocket, forming a π–π interaction with Y34 and W98, while Q484’s side chain had to shift outward to avoid electrostatic repulsion (Figure 10f). This alteration may explain the significant change in RBD orientation. Despite the PPD results suggesting numerous interactions between Kappa’s RBD and the 2B04 antibody, the reduced interaction surface area indicates that such a complex would likely be unstable in further MD simulations. AlphaFold predicted no interactions for this complex, aligning well with the experimentally observed low affinity.

Another intriguing observation regarding AlphaFold 3.0 emerged in its prediction of protein structures. Following the completion of this study, the Cryo-EM structure of the SARS-CoV-2 Omicron JN.1 spike protein (PDB ID: 8Y5J) was released, enabling a direct comparison between the homology model built here and crystallographic data. The homology modeling approach used here was successfully applied in previous studies [48,50] to predict the 3D structures of various SARS-CoV-2 variants, including Alpha, Beta, Gamma, Delta, Epsilon, and Omicron subvariants, with RMSD values consistently below 2 Å when aligned with reference crystallographic structures. As tested after the completion of all calculations, for the Omicron JN.1 homology model, alignment with the corresponding reference structure resulted in RMSD values of 3.7120 Å, 3.2412 Å, and 3.6317 Å for the RBDs of chains A, B, and C, respectively. These deviations raise concerns about the model’s accuracy, particularly in the context of protein–protein docking predictions, where such discrepancies may have a significant impact, although their influence on molecular dynamics simulations is somewhat less pronounced. To explore an alternative, we employed AlphaFold 3.0 to predict the structure of the Omicron JN.1 RBD. The resulting RMSD values for the backbone ranged from 2.4720 Å to 2.6626 Å across five predicted clusters when compared to reference chain A, from 2.9829 Å to 3.1695 Å for chain B, and from 2.2332 Å to 2.4172 Å for chain C. These values indicate that AlphaFold 3.0 outperforms conventional homology modeling in predicting the RBD structure. However, further testing is necessary to fully evaluate this observation, which lies beyond the scope of the present study.

## 5. Conclusions

This study comprehensively evaluated the efficacy of three computational approaches used to study ligand–protein interactions—forced placement with molecular dynamics simulations, protein–protein docking, and AlphaFold complex structure prediction—in predicting 2B04 antibody neutralization of SARS-CoV-2 variants. Our comparative analysis demonstrated distinct advantages and limitations of each approach, shedding light on their predictive power and alignment with experimental immune escape data.

The forced placement method, while capable of providing insights into antibody–RBD interactions, struggled with accurately predicting immune escape for complex variants such as Epsilon and Kappa. This approach’s reliance on initial structure guesses led to challenges in accurately capturing the dynamic nature of these interactions, resulting in a limited description of the experimental immune escape data.

Despite requiring less computational time, protein docking was highly sensitive to the initial antibody structure and suffered from rigid-body assumptions that introduced inaccuracies. Although this method showed slightly better correlations when assessing immune escape potential using RMSD and protein–protein interaction (PPI) Scores, it lacked the robustness for comprehensive predictions.

In contrast, AlphaFold demonstrated higher accuracy and efficiency, showing a better correlation with experimental neutralization data across tested variants. Moreover, AlphaFold’s proficiency in accurately predicting the RBD’s secondary structure for emerging variants suggests significant promise for its application in future studies.

Our findings underscore the importance of employing a multi-faceted computational strategy to address the rapid mutation rate of SARS-CoV-2 and its implications for immune escape. This knowledge is critical for improving the predictive accuracy of vaccine efficacy against new and emerging viral subvariants, thereby aiding in the development of more effective immunization strategies.

## Figures and Tables

**Table 1:** Protein interaction analysis of the final RBD/antibody complexes after 200 ns MD simulation clustering.

Variant	#HB	#Salt Bridges	#*p* Stacking	#vdW Clashes	The Sum of Surface Complementarity	The Sum of Buried SASA
WT	10	0	1	0	10.44	9.625
Alpha	8	1	1	3	13.61	9.269
Beta	6	0	0	2	13.63	13.891
Gamma	7	0	1	1	10.75	7.83
Delta	7	0	2	0	8.19	7.71
Kappa	8	0	1	2	10.73	8.128
Epsilon	2	0	0	0	9.87	7.162
Epsilon *	4	0	1	0	9.03	7.335
Omicron BA.1	6	0	1	0	9.42	7.854
Omicron JN.1	4	0	0	2	8.32	7.971

* The most populated cluster of the 200 ns simulation, with the initial structure derived from the fifth most populated cluster of the 100 ns preliminary MD simulation.

**Table 2:** Results of protein–protein docking between the antibody and the WT RBD utilizing five different settings.

Setting	Default		Masked non-CDR		Masked non-CDR + 1 Restrained Residue (10 Å)		Masked non-CDR + 2 Restrained Residue (10 Å)		Masked non-CDR + 2 Restrained Residue (20 Å)	
Cluster	PIPER Pose Score	RMSD	PIPER Pose Score	RMSD	PIPER Pose Score	RMSD	PIPER Pose Score	RMSD	PIPER Pose Score	RMSD
Pose 1	−457.198 ^a^	7.646 ^a^	−293.516	27.978	−333.482	8.291	−284.895	13.655	−285.438 ^a^	6.091 ^a^
Pose 2	−293.516	27.978	−440.06	6.454	−340.992	41.611	−150.677 ^a^	6.621 ^a^	−327.952	7.772
Pose 3	−388.143	12.809	−359.293	24.632	−328.815	32.267	−150.317	8.407	−329.829	20.186
Pose 4	−266.848	25.264	−209.244 ^a^	3.63 ^a^	−375.115	16.898	−260.251	22.411	−185.388	16.432
Pose 5	−258.313	30.798	−349.946	29.871	−241.792	35.909	−242.231	12.895	−166.638	17.425
Pose 6	−387.458	31.574	−267.746	30.229	−297.668	21.997	−54.547	11.938	−82.759	18.215
Pose 7	−250.088	20.188	−406.003	30.684	−458.749	42.156	−145.266	25.732	−141.188	15.38
Pose 8	−250.482	33.641	−175.706	24.512	−256.155	13.457	−96.679	8.31	−14.671	16.466
Pose 9	−210.379	23.625	−371.484	14.758	−261.563	15.539	−113.486	22.374	−181.984	8.23
Pose 10	−401.988	30.996	−302.371	32.238	−276.454	30.954			−230.137	21.929
Pose 11	−207.697	26.078	−309.833	18.977	−190.658 ^a^	7.616 ^a^			−95.969	25.936
Pose 12	−221.059	40.109	−222.226	30.54	−348.832	39.941			−222.186	30.122
Pose 13	−271.411	31.393	−206.757	23.489	−235.75	24.04			−218.579	26.757
Pose 14	−322.635	31.796	−219.762	27.718	−218.887	46.816			−69.551	11.574
Pose 15	−142.35	21.944	−339.772	26.577	−161.378	24.409			−33.784	21.102
Pose 16	−317.152	14.218	−250.482	33.641	−294.323	22.275			−53.397	30.643
Pose 17	−219.762	27.718	−251.206	26.989	−86.457	18.959			−134.117	14.686
Pose 18	−142.251	30.677	−212.289	30.442	−391.134	17.908			−172.463	29.637
Pose 19	−309.501	10.353	−247.819	30.659	−360.8	40.092			−93.918	24.066
Pose 20	−335.719	30.454	−260.417	30.23	−225.572	41.739				
Pose 21	−260.176	20.924	−263.305	24.746	−222.689	36.605				
Pose 22	−226.819	13.679	−235.555	21.632	−9.888	22.905				
Pose 23	−223.198	23.523	−264.263	21.063	−291.837	24.137				
Pose 24	−206.177	22.799	−366.239	11.69	−164.386	31.189				
Pose 25	−169.366	33.549	−267.54	24.5						
Pose 26	−215.471	23.003	−268.67	30.412						
Pose 27	−173.55	20.441	−281.905	21.807						
Pose 28	−187.569	28.539	−187.569	28.539						
Pose 29	−231.283	28.295	−293.907	27.269						
Pose 30	−195.066	32.712	−278.004	29.488						
Wall time ^b^	0H 21′ 36″		0H 22′ 44″		0H 21′ 15″		0H 05′ 56″		0H 44′ 47″	

^a^ Pose with the lowest RMSD relative to the reference crystallographic structure. ^b^ Number of processes 12; Linux-x86_64; Resources: Z8G4 2.4 GHz Intel Xeon Silver 4214R 12-Core 64GB 2933 MHz DDR4 ECC Registered RAM NVIDIA Quadro RTX A5000 1TB.

**Table 3:** Protein interaction analysis of the lowest RMSD-value poses from protein–protein docking.

Variant	#HB	#Salt Bridges	#*p* Stacking	#vdW Clashes	Surface Complementarity	Buried SASA
WT	6	0	0	23	8.92	8.491
Alpha	1	0	0	119	9.01	9.95
Beta	1	0	1	93	7.76	10.44
Gamma	2	0	1	74	7.18	9.297
Delta	0	0	0	76	7.31	10.365
Kappa	2	0	4	77	6.09	10.21
Epsilon	0	0	1	84	6.09	6.754
Omicron BA.1	1	0	0	66	10.71	12.984
Omicron JN.1	0	0	0	59	7.41	13.464

## Data Availability

The dataset is available upon request from the authors.

## Supplementary Materials

The following supporting information can be downloaded at: https://www.mdpi.com/article/10.3390/cimb46110745/s1, the clustering analysis for MD simulations (Tables S1–S12, Figure S1), the results of a protein–protein docking (Table S13) and sequences of the 2B04 antibody and mutant’s RBDs (Table S14).
