# Supplementary material for: Benchmark Investigation of SARS-CoV-2 Mutants’ Immune Escape with 2B04 Murine Antibody: A Step Towards Unraveling a Larger Picture
Source: Curr Issues Mol Biol. 2024 Nov 6;46(11):12550–73. doi: 10.3390/cimb46110745 (PMC11592782; doi:10.3390/cimb46110745)
Supplement: Supplementary file 1 [file cimb-46-00745-s001.zip › cimb-3203884-supplementary.pdf]

Benchmark Investigation of SARS-CoV-2 Mutants' Immune Escape with 2B04 Murine Antibody: A Step Towards Unravelling a Larger Picture

Karina Kapusta [1,*], Allyson McGowan [1], Santanu Banerjee [1], Jing Wang [2], Wojciech Kolodziejczyk[2] and Jerzy Leszczynski [2]

[1] Department of Chemistry and Physics, Tougaloo College, Tougaloo, MS, 39174, USA; kkapusta@tougaloo.edu
[2] Department of Chemistry, Physics and Atmospheric Sciences, Jackson State University, Jackson, MS, 39217, USA; jerzy@icnanotox.org
* Correspondence: jerzy@icnanotox.org; kkapusta@tougaloo.edu.

Supporting Information

Table S1. Clustering analysis for 100 ns preliminary Molecular Dynamics Simulation.

Cluster	Number of Frames in Cluster	Frame Index	Chemical Time, ps	RMSD, Å
		**Wild Type**		
**Cluster 1**	44	675	67501.35	2.107
**Cluster 2**	37	234	23400.468	1.927
**Cluster 3**	35	454	45400.908	2.011
**Cluster 4**	27	832	83201.664	2.152
**Cluster 5**	26	736	73601.472	1.980
**Cluster 6**	26	397	39700.794	1.975
**Cluster 7**	25	581	58101.162	2.040
**Cluster 8**	25	514	51401.028	1.990
**Cluster 9**	24	482	48200.964	1.889
**Cluster 10**	24	247	24700.494	2.118
		**Alpha**		
**Cluster 1**	31	645	64501.29	1.833
**Cluster 2**	25	831	83101.662	1.836
**Cluster 3**	23	657	65701.314	2.188
**Cluster 4**	23	563	56301.126	1.982
**Cluster 5**	23	164	16400.328	2.246
**Cluster 6**	21	877	87701.754	2.022
**Cluster 7**	21	649	64901.298	1.878
**Cluster 8**	20	864	86401.728	2.087
**Cluster 9**	20	688	68801.376	2.211
**Cluster 10**	20	296	29600.592	2.072
		**Beta**		
**Cluster 1**	78	862	86201.724	3.295
**Cluster 2**	62	787	78701.574	3.298

**Cluster 3**	60	745	74501.49	2.923
**Cluster 4**	55	488	48800.976	3.405
**Cluster 5**	48	947	94701.894	3.129
**Cluster 6**	42	422	42200.844	3.209
**Cluster 7**	34	957	95701.914	3.172
**Cluster 8**	31	998	99801.996	3.304
**Cluster 9**	31	113	11300.226	2.378
**Cluster 10**	30	370	37000.74	3.380
**Gamma**				
**Cluster 1**	35	485	48500.97	1.998
**Cluster 2**	26	401	40100.802	1.927
**Cluster 3**	25	759	75901.518	2.166
**Cluster 4**	25	391	39100.782	2.083
**Cluster 5**	22	737	73701.474	2.283
**Cluster 6**	21	633	63301.266	2.009
**Cluster 7**	20	861	86101.722	2.156
**Cluster 8**	20	591	59101.182	1.915
**Cluster 9**	20	438	43800.876	1.642
**Cluster 10**	20	119	11900.238	2.258
**Delta**				
**Cluster 1**	33	797	79701.594	2.042
**Cluster 2**	31	698	69801.396	1.910
**Cluster 3**	28	490	49000.98	1.949
**Cluster 4**	25	950	95001.9	2.187
**Cluster 5**	24	1000	100000.008	2.369
**Cluster 6**	23	878	87801.756	2.039
**Cluster 7**	23	746	74601.492	2.008
**Cluster 8**	22	221	22100.442	1.988
**Cluster 9**	21	920	92001.84	2.352
**Cluster 10**	20	666	66601.332	2.118
**Epsilon**				
**Cluster 1**	50	912	91201.824	2.706
**Cluster 2**	42	624	62401.248	2.831
**Cluster 3**	42	572	57201.144	2.755
**Cluster 4**	42	148	14800.296	2.840
**Cluster 5**	41	398	39800.796	2.339
**Cluster 6**	37	778	77801.556	3.013
**Cluster 7**	37	620	62001.24	3.014
**Cluster 8**	36	729	72901.458	2.914
**Cluster 9**	36	275	27500.55	2.682
**Cluster 10**	35	651	65101.302	2.897
**Kappa**				
**Cluster 1**	38	208	20800.416	2.135
**Cluster 2**	36	252	25200.504	1.792
**Cluster 3**	35	771	77101.542	2.049
**Cluster 4**	35	322	32200.644	2.278
**Cluster 5**	32	331	33100.662	1.877

**Cluster 6**	31	525	52501.05	2.018
**Cluster 7**	31	345	34500.69	1.847
**Cluster 8**	30	187	18700.374	1.780
**Cluster 9**	29	247	24700.494	1.971
**Cluster 10**	26	791	79101.582	1.888
		**Omicron BA.1**		
**Cluster 1**	52	185	18500.37	2.118
**Cluster 2**	42	515	51501.03	1.871
**Cluster 3**	34	529	52901.058	2.260
**Cluster 4**	28	765	76501.53	2.125
**Cluster 5**	28	726	72601.452	2.160
**Cluster 6**	27	675	67501.35	2.025
**Cluster 7**	23	585	58501.17	2.123
**Cluster 8**	22	664	66401.328	1.994
**Cluster 9**	22	596	59601.192	2.183
**Cluster 10**	21	867	86701.734	2.119
		**Omicron JN.1**		
**Cluster 1**	39	504	50401.008	2.082
**Cluster 2**	39	293	29300.586	2.363
**Cluster 3**	32	941	94101.882	2.432
**Cluster 4**	30	731	73101.462	2.430
**Cluster 5**	29	877	87701.754	2.441
**Cluster 6**	26	286	28600.572	2.206
**Cluster 7**	25	667	66701.334	2.121
**Cluster 8**	25	616	61601.232	2.511
**Cluster 9**	24	779	77901.558	2.435
**Cluster 10**	24	683	68301.366	2.490

Table S2. Clustering analysis for 200 ns Molecular Dynamics Simulation.

Cluster	Number of Frames in Cluster	Frame Index	Chemical Time, ps	RMSD, Å
		**Wild Type**		
**Cluster 1**	35	1721	172103.442	2.143
**Cluster 2**	34	1441	144102.882	2.070
**Cluster 3**	33	1921	192103.842	2.039
**Cluster 4**	32	761	76101.522	2.090
**Cluster 5**	31	1969	196903.938	1.882
**Cluster 6**	30	1604	160403.208	2.060
**Cluster 7**	29	1766	176603.532	1.759
**Cluster 8**	27	1536	153603.072	1.939
**Cluster 9**	27	172	17200.344	2.009
**Cluster 10**	26	1715	171503.43	2.105
		**Alpha**		
**Cluster 1**	71	762	76201.524	1.998
**Cluster 2**	49	1655	165503.31	2.051
**Cluster 3**	45	1369	136902.738	2.003
**Cluster 4**	39	1286	128602.572	1.869

**Cluster 5**	37	1628	162803.256	2.002
**Cluster 6**	36	1259	125902.518	1.739
**Cluster 7**	36	1149	114902.298	2.206
**Cluster 8**	35	1026	102602.052	2.005
**Cluster 9**	35	971	97101.942	1.993
**Cluster 10**	35	432	43200.864	1.708
		**Beta**		
**Cluster 1**	41	1070	107002.14	3.098
**Cluster 2**	39	1040	104002.08	3.304
**Cluster 3**	38	1144	114402.288	3.000
**Cluster 4**	32	858	85801.716	3.151
**Cluster 5**	32	210	21000.42	2.893
**Cluster 6**	31	1841	184103.682	3.681
**Cluster 7**	31	1009	100902.018	3.436
**Cluster 8**	30	1906	190603.812	3.336
**Cluster 9**	30	1196	119602.392	3.447
**Cluster 10**	29	1300	130002.6	3.071
		**Gamma**		
**Cluster 1**	60	425	42500.85	1.962
**Cluster 2**	43	1329	132902.658	1.980
**Cluster 3**	38	489	48900.978	2.085
**Cluster 4**	38	241	24100.482	2.221
**Cluster 5**	34	255	25500.51	1.882
**Cluster 6**	34	153	15300.306	2.006
**Cluster 7**	33	1759	175903.518	2.488
**Cluster 8**	33	390	39000.78	1.948
**Cluster 9**	32	1356	135602.712	1.942
**Cluster 10**	32	165	16500.33	1.929
		**Delta**		
**Cluster 1**	36	837	83701.674	1.952
**Cluster 2**	34	1453	145302.906	1.990
**Cluster 3**	34	979	97901.958	1.864
**Cluster 4**	31	1155	115502.31	1.950
**Cluster 5**	30	1607	160703.214	2.017
**Cluster 6**	30	386	38600.772	2.145
**Cluster 7**	29	1153	115302.306	1.997
**Cluster 8**	28	1409	140902.818	2.227
**Cluster 9**	27	653	65301.306	1.790
**Cluster 10**	26	1495	149502.99	2.089
		**Epsilon**		
**Cluster 1**	71	496	49600.992	2.764
**Cluster 2**	52	764	76401.528	2.974
**Cluster 3**	51	876	87601.752	2.662
**Cluster 4**	49	16	1600.032	3.016
**Cluster 5**	48	914	91401.828	2.944
**Cluster 6**	46	1580	158003.16	3.130
**Cluster 7**	46	342	34200.684	2.898

**Cluster 8**	45	1320	132002.64	2.965
**Cluster 9**	44	780	78001.56	3.184
**Cluster 10**	42	546	54601.092	2.936
**Epsilon***				
**Cluster 1**	51	567	56701.134	3.045
**Cluster 2**	49	597	59701.194	2.960
**Cluster 3**	48	421	42100.842	3.045
**Cluster 4**	48	416	41600.832	3.202
**Cluster 5**	46	1347	134702.694	3.462
**Cluster 6**	45	548	54801.096	3.314
**Cluster 7**	43	1696	169603.392	3.297
**Cluster 8**	43	1206	120602.412	3.080
**Cluster 9**	43	267	26700.534	2.919
**Cluster 10**	39	1146	114602.292	2.807
**Kappa**				
**Cluster 1**	48	1447	144702.894	1.933
**Cluster 2**	41	1744	174403.488	1.771
**Cluster 3**	40	1252	125202.504	1.715
**Cluster 4**	39	371	37100.742	1.888
**Cluster 5**	36	847	84701.694	2.028
**Cluster 6**	34	61	6100.122	2.167
**Cluster 7**	32	870	87001.74	2.104
**Cluster 8**	32	368	36800.736	2.159
**Cluster 9**	31	1600	160003.2	1.913
**Cluster 10**	31	1575	157503.15	2.305
**Omicron BA.1**				
**Cluster 1**	62	1060	106002.12	2.188
**Cluster 2**	56	857	85701.714	1.950
**Cluster 3**	47	1526	152603.052	2.151
**Cluster 4**	45	1113	111302.226	2.214
**Cluster 5**	41	1601	160103.202	2.172
**Cluster 6**	39	371	37100.742	1.885
**Cluster 7**	38	767	76701.534	1.810
**Cluster 8**	34	1537	153703.074	1.968
**Cluster 9**	34	422	42200.844	1.764
**Cluster 10**	33	1696	169603.392	2.195
**Omicron JN.1**				
**Cluster 1**	38	571	57101.142	1.796
**Cluster 2**	38	71	7100.142	2.233
**Cluster 3**	37	1907	190703.814	3.247
**Cluster 4**	32	1448	144802.896	1.996
**Cluster 5**	32	1332	133202.664	2.355
**Cluster 6**	32	1055	105502.11	1.970
**Cluster 7**	32	269	26900.538	1.913
**Cluster 8**	31	549	54901.098	2.371
**Cluster 9**	28	915	91501.83	2.118
**Cluster 10**	28	676	67601.352	1.953

* The initial structure for the Epsilon trajectory was derived from the most populated cluster of the 100 ns preliminary Epsilon MD simulation, while the initial structure for the Epsilon* was the fifth most populated cluster (Table S1) with the smallest RMSD of 100 ns preliminary Epsilon MD simulation.

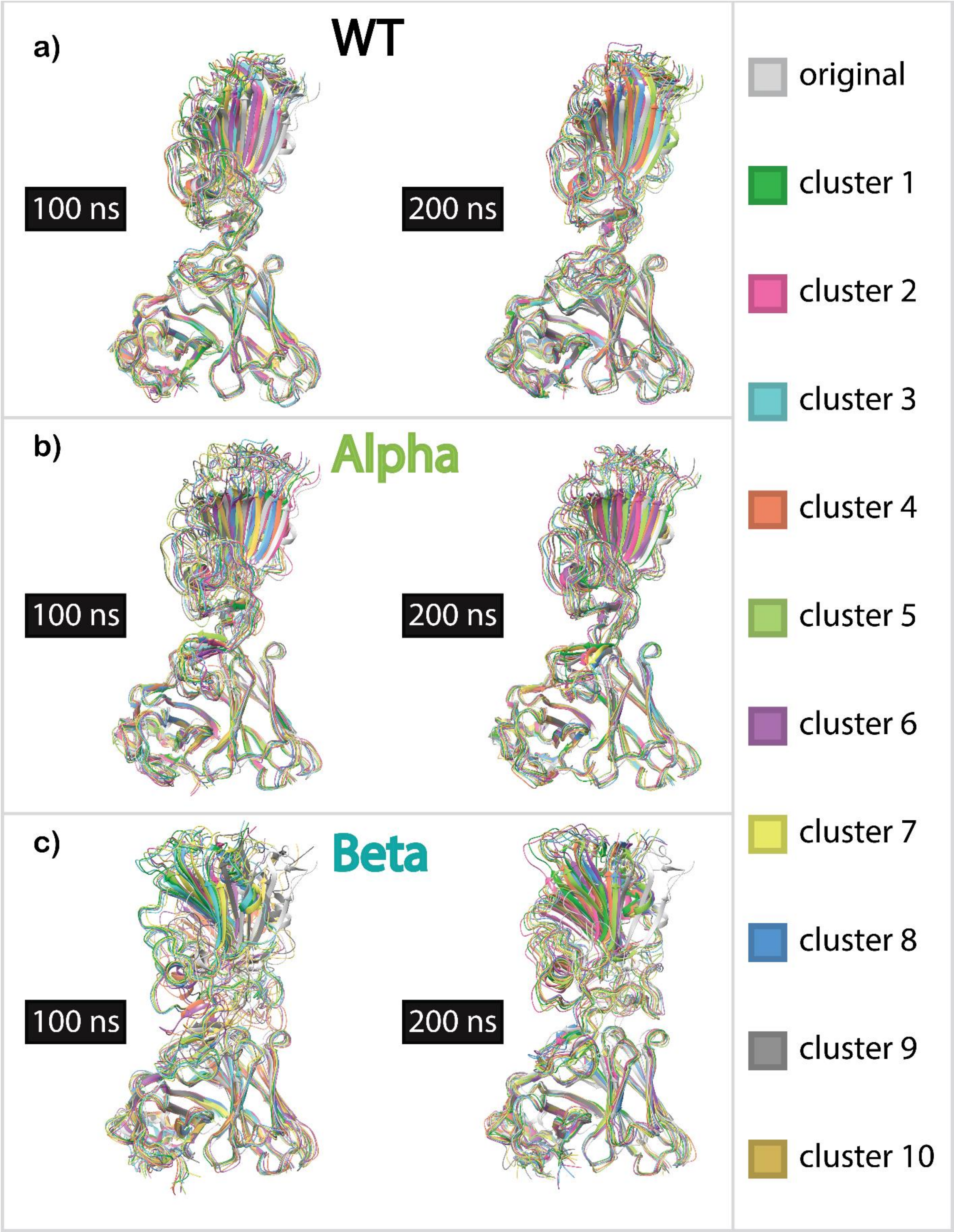

Figure S1. 10 most populated clusters results of Molecular Dynamics simulations for initial 100 ns run and 200 ns run of complexes: a) – Wild Type; b) – Alpha; c) – Beta; d) – Gamma; e) – Delta; f) – Kappa; g) – Epsilon; h) – Omicron BA.1; i) – Omicron JN.1.

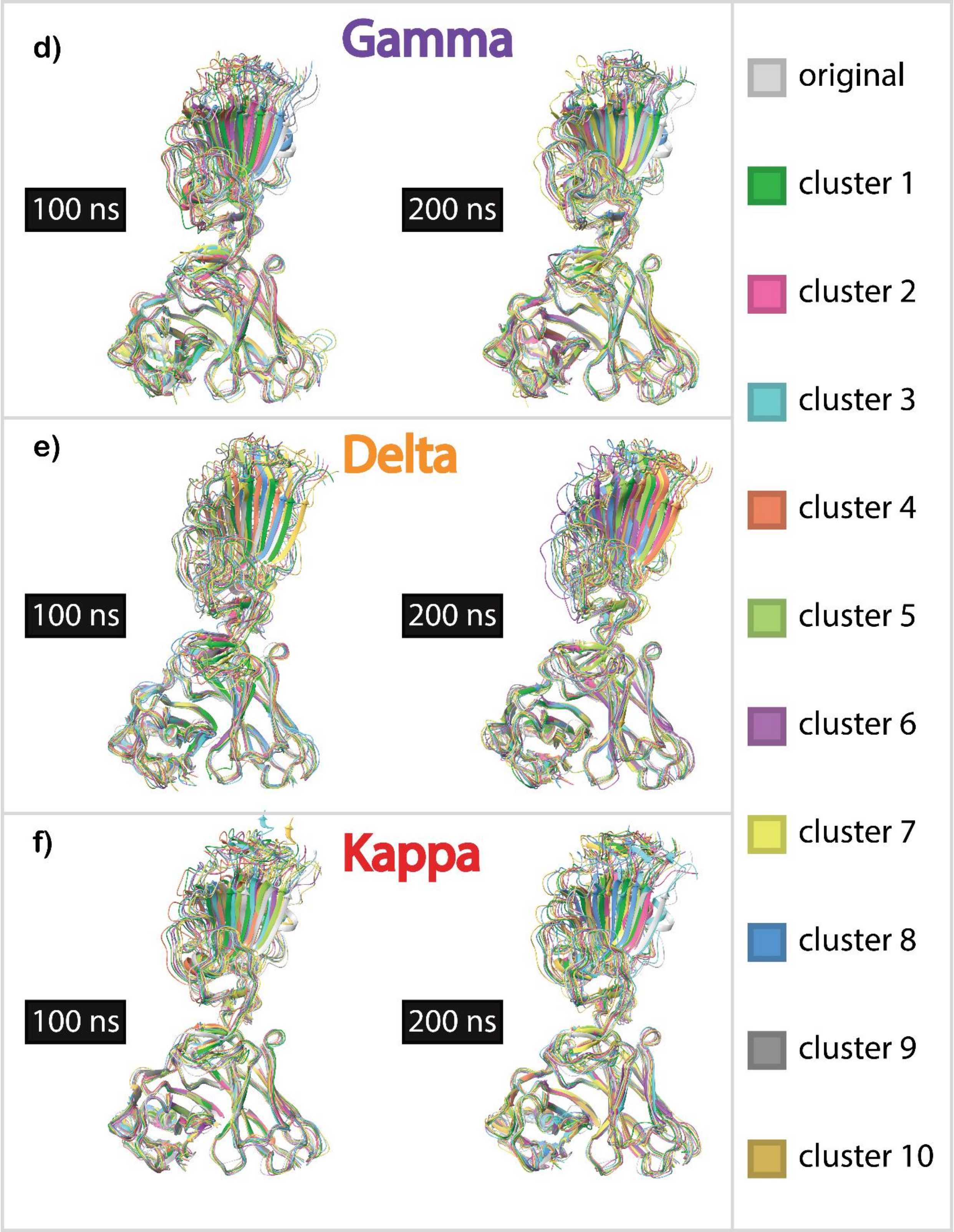

Figure S1 (continued).

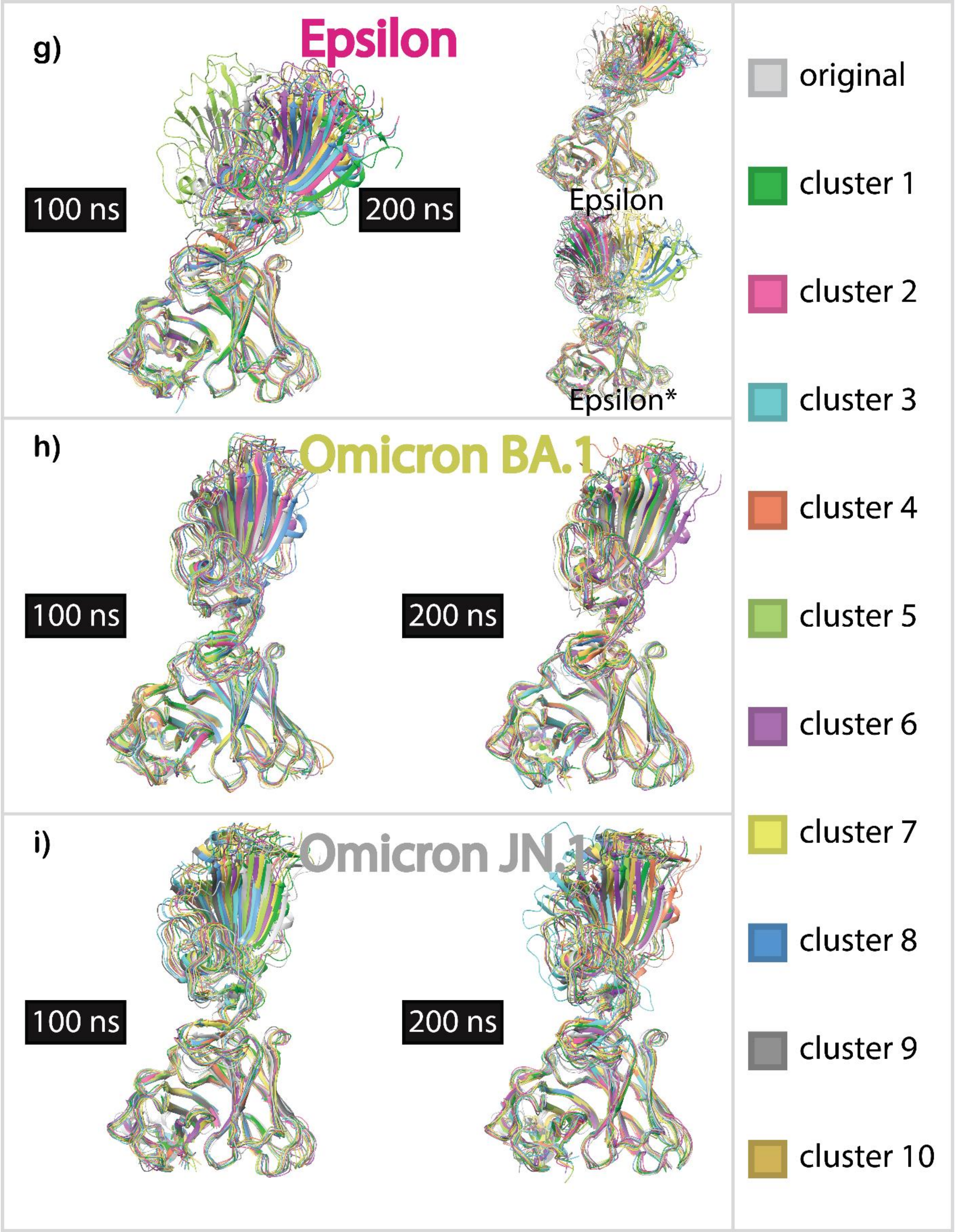

Figure S1 (continued).

Table S3. Protein-Protein Interactions for the Wild Type RBD/2B04.

Residue	Closest	Distance	Specific Interactions	# HB	# Salt Bridges	# Pi Stacking	# Disulfides	# vdW Clash	Surface Complementarity	Buried SASA
A:445:Val				0	0	0	0	0	0	3.50%
A:446:Gly				0	0	0	0	0	0	9.00%
A:449:Tyr	H:28:Ser	3.6 A		0	0	0	0	0	0.69	66.50%
A:452:Leu				0	0	0	0	0	0.87	27.80%
A:453:Tyr				0	0	0	0	0	0	0.80%
A:455:Leu	H:101:Tyr	3.4 A		0	0	0	0	0	0.85	80.90%
A:456:Phe	H:101:Tyr	3.7 A		0	0	0	0	0	0.21	28.20%
A:473:Tyr				0	0	0	0	0	0.06	2.90%
A:483:Val				0	0	0	0	0	0	4.20%
A:484:Glu	H:56:Gly<br>H:54:Gly<br>H:55:Gly<br>H:53:Thr<br>H:52:Trp	2.7 A<br>2.9 A<br>3.1 A<br>3.3 A<br>3.6 A	1x hb to H:54:Gly<br>1x hb to H:55:Gly<br>1x hb to H:56:Gly	3	0	0	0	0	0.75	84.40%
A:485:Gly	H:100:Tyr<br>H:52:Trp	3.2 A<br>3.6 A		0	0	0	0	0	0.69	97.90%
A:486:Phe	H:100:Tyr<br>H:58:Asn	3.5 A<br>4.0 A		0	0	0	0	0	0.55	39.10%
A:487:Asn	H:100:Tyr	2.9 A	1x hb to H:100:Tyr	1	0	0	0	0	0.77	25.50%
A:488:Cys	H:100:Tyr	3.0 A	1x hb to H:100:Tyr	1	0	0	0	0	0.8	93.60%
A:489:Tyr	H:100:Tyr<br>H:101:Tyr	2.9 A<br>3.3 A	1x hb, 1x pi stack to H:100:Tyr	1	0	1	0	0	0.83	88.20%
A:490:Phe	H:54:Gly<br>H:53:Thr	3.8 A<br>4.0 A		0	0	0	0	0	0.54	39.20%
A:491:Pro				0	0	0	0	0	0.36	34.40%
A:492:Leu	H:30:Ile	3.7 A		0	0	0	0	0	0.83	56.10%
A:493:Gln	H:101:Tyr<br>H:31:Asn	3.4 A<br>3.5 A		0	0	0	0	0	0.82	71.80%
A:494:Ser	H:31:Asn<br>H:28:Ser<br>H:30:Ile	2.8 A<br>3.3 A<br>3.7 A	1x hb to H:28:Ser<br>3x hb to H:31:Asn	4	0	0	0	0	0.82	92.40%
A:498:Gln				0	0	0	0	0	0	13.70%
A:500:Thr				0	0	0	0	0	0	2.40%

H:1:Gln				0	0	0	0	0	0	3.60%
H:25:Ser				0	0	0	0	0	0	4.20%
H:26:Gly				0	0	0	0	0	0.49	50.50%
H:27:Phe				0	0	0	0	0	0.46	52.10%
H:28:Ser	A:494:Ser<br>A:449:Tyr	3.3 A<br>3.6 A	1x hb to A:494:Ser	1	0	0	0	0	0.87	88.80%
H:30:Ile	A:492:Leu<br>A:494:Ser	3.7 A<br>3.7 A		0	0	0	0	0	0.83	76.90%
H:31:Asn	A:494:Ser<br>A:493:Gln	2.8 A<br>3.5 A	3x hb to A:494:Ser	3	0	0	0	0	0.79	70.10%
H:52:Trp	A:485:Gly<br>A:484:Glu	3.6 A<br>3.6 A		0	0	0	0	0	0.7	52.40%
H:53:Thr	A:484:Glu<br>A:490:Phe	3.3 A<br>4.0 A		0	0	0	0	0	0.38	95.20%
H:54:Gly	A:484:Glu<br>A:490:Phe	2.9 A<br>3.8 A	1x hb to A:484:Glu	1	0	0	0	0	0.85	67.90%
H:55:Gly	A:484:Glu	3.1 A	1x hb to A:484:Glu	1	0	0	0	0	0.83	5.20%
H:56:Gly	A:484:Glu	2.7 A	1x hb to A:484:Glu	1	0	0	0	0	0.73	58.70%
H:57:Thr				0	0	0	0	0	0	3.60%
H:58:Asn	A:486:Phe	4.0 A		0	0	0	0	0	0.5	58.70%
H:76:Ser				0	0	0	0	0	0	42.00%
H:99:Asp				0	0	0	0	0	0	3.20%
H:100:Tyr	A:489:Tyr<br>A:487:Asn<br>A:488:Cys<br>A:485:Gly<br>A:486:Phe	2.9 A<br>2.9 A<br>3.0 A<br>3.2 A<br>3.5 A	1x hb to A:487:Asn<br>1x hb to A:488:Cys<br>1x hb, 1x pi stack to A:489:Tyr	3	0	1	0	0	0.82	86.30%
H:101:Tyr	A:489:Tyr<br>A:455:Leu<br>A:493:Gln<br>A:456:Phe	3.3 A<br>3.4 A<br>3.4 A<br>3.7 A		0	0	0	0	0	0.84	68.70%
H:105:Tyr				0	0	0	0	0	0	5.10%
L:93:Trp				0	0	0	0	0	0	17.20%
L:95:Asn				0	0	0	0	0	0	4.20%
L:96:Asn				0	0	0	0	0	0	11.90%

Table S4. Protein-Protein Interactions for the Alpha RBD/2B04.

Residue	Closest	Distance	Specific Interactions	# HB	# Salt Bridges	# Pi Stacking	# Disulfides	# vdW Clash	Surface Complementarity	Buried SASA
A:445:Val				0	0	0	0	0	0.65	18.30%
A:446:Gly	H:26:Gly<br>H:27:Phe	3.5 A<br>3.8 A		0	0	0	0	0	0.86	69.70%
A:448:Asn				0	0	0	0	0	0	7.40%
A:449:Tyr	H:74:Ser<br>H:30:Ile	3.2 A<br>3.4 A		0	0	0	0	0	0.83	54.00%
A:452:Leu	H:30:Ile	3.8 A		0	0	0	0	0	0.88	30.60%
A:455:Leu				0	0	0	0	0	0.64	10.30%
A:472:Ile	H:55:Gly	3.9 A		0	0	0	0	0	0.59	26.00%
A:481:Asn	H:58:Asn	3.2 A		0	0	0	0	0	0.86	30.20%
A:482:Gly	H:52:Trp<br>H:58:Asn	3.5 A<br>3.9 A		0	0	0	0	0	0.91	63.30%
A:483:Val	H:54:Gly<br>H:52:Trp<br>H:100:Tyr<br>H:53:Thr	3.1 A<br>3.4 A<br>3.5 A<br>4.0 A	1x clash to H:52:Trp	0	0	0	0	1	0.75	91.50%
A:484:Glu	H:98:Lys<br>H:100:Tyr<br>H:52:Trp	2.9 A<br>3.4 A<br>3.7 A	1x hb, 1x salt bridge to H:98:Lys<br>2x clash to H:100:Tyr	1	1	0	0	2	0.82	87.50%
A:485:Gly	H:100:Tyr	2.8 A	1x hb to H:100:Tyr	1	0	0	0	0	0.7	73.60%
A:486:Phe	L:34:Tyr	3.9 A		0	0	0	0	0	0	14.50%
A:487:Asn				0	0	0	0	0	0.43	2.00%
A:488:Cys	H:100:Tyr	3.0 A	1x hb to H:100:Tyr	1	0	0	0	0	0.67	10.50%
A:489:Tyr	H:100:Tyr<br>H:101:Tyr	3.4 A<br>3.6 A	1x pi stack to H:101:Tyr	0	0	1	0	0	0.87	45.90%
A:490:Phe	H:101:Tyr<br>H:54:Gly<br>H:53:Thr	2.9 A<br>3.5 A<br>3.7 A	1x hb to H:101:Tyr	1	0	0	0	0	0.72	79.20%
A:492:Leu	H:30:Ile	3.9 A		0	0	0	0	0	0.74	12.60%
A:493:Gln	H:101:Tyr<br>H:31:Asn<br>H:30:Ile	3.4 A<br>3.4 A<br>3.5 A		0	0	0	0	0	0.85	87.20%
A:494:Ser	H:31:Asn<br>H:28:Ser<br>H:30:Ile	2.7 A<br>2.9 A<br>4.0 A	1x hb to H:28:Ser<br>3x hb to H:31:Asn	4	0	0	0	0	0.84	93.60%
A:495:Tyr				0	0	0	0	0	0	16.80%

A:496:Gly				0	0	0	0	0	0	2.20%
H:1:Gln				0	0	0	0	0	0	1.60%
H:25:Ser				0	0	0	0	0	0	12.20%
H:26:Gly	A:446:Gly	3.5 A		0	0	0	0	0	0.76	68.80%
H:27:Phe	A:446:Gly	3.8 A		0	0	0	0	0	0.85	55.70%
H:28:Ser	A:494:Ser	2.9 A	1x hb to<br>A:494:Ser	1	0	0	0	0	0.8	77.80%
H:29:Leu				0	0	0	0	0	0	35.80%
H:30:Ile	A:449:Tyr<br>A:493:Gln<br>A:452:Leu<br>A:492:Leu<br>A:494:Ser	3.4 A<br>3.5 A<br>3.8 A<br>3.9 A<br>4.0 A		0	0	0	0	0	0.84	93.20%
H:31:Asn	A:494:Ser<br>A:493:Gln	2.7 A<br>3.4 A	3x hb to<br>A:494:Ser	3	0	0	0	0	0.83	70.10%
H:33:Ala				0	0	0	0	0	0.15	33.80%
H:52:Trp	A:483:Val<br>A:482:Gly<br>A:484:Glu	3.4 A<br>3.5 A<br>3.7 A	1x clash to<br>A:483:Val	0	0	0	0	1	0.85	85.40%
H:53:Thr	A:490:Phe<br>A:483:Val	3.7 A<br>4.0 A		0	0	0	0	0	0.73	92.30%
H:54:Gly	A:483:Val<br>A:490:Phe	3.1 A<br>3.5 A		0	0	0	0	0	0.82	85.10%
H:55:Gly	A:472:Ile	3.9 A		0	0	0	0	0	0.59	48.40%
H:58:Asn	A:481:Asn<br>A:482:Gly	3.2 A<br>3.9 A		0	0	0	0	0	0.82	42.70%
H:73:Asn				0	0	0	0	0	0.44	32.50%
H:74:Ser	A:449:Tyr	3.2 A		0	0	0	0	0	0.79	41.30%
H:98:Lys	A:484:Glu	2.9 A	1x hb, 1x salt<br>bridge to<br>A:484:Glu	1	1	0	0	0	0.39	59.80%
H:100:Tyr	A:485:Gly<br>A:488:Cys<br>A:484:Glu<br>A:489:Tyr<br>A:483:Val	2.8 A<br>3.0 A<br>3.4 A<br>3.4 A<br>3.5 A	2x clash to<br>A:484:Glu<br>1x hb to<br>A:485:Gly<br>1x hb to<br>A:488:Cys	2	0	0	0	2	0.81	82.90%
H:101:Tyr	A:490:Phe<br>A:493:Gln<br>A:489:Tyr	2.9 A<br>3.4 A<br>3.6 A	1x pi stack to<br>A:489:Tyr<br>1x hb to<br>A:490:Phe	1	0	1	0	0	0.75	67.30%
H:105:Tyr				0	0	0	0	0	0.69	33.80%
L:32:Ser				0	0	0	0	0	0	6.60%
L:34:Tyr	A:486:Phe	3.9 A		0	0	0	0	0	0	43.40%
L:93:Trp				0	0	0	0	0	0	9.00%
L:96:Asn				0	0	0	0	0	0	14.00%
L:98:Trp				0	0	0	0	0	0	24.30%

Table S5. Protein-Protein Interactions for the Beta RBD/2B04.

Residue	Closest	Distance	Specific Interactions	# HB	# Salt Bridges	# Pi Stacking	# Disulfides	# vdW Clash	Surface Complementarity	Buried SASA
A:403:Arg				0	0	0	0	0	0.55	18.50%
A:445:Val				0	0	0	0	0	0	0.50%
A:446:Gly	H:74:Ser<br>H:73:Asn	3.9 A<br>3.9 A		0	0	0	0	0	0.58	50.40%
A:449:Tyr	H:73:Asn<br>H:28:Ser<br>H:30:Ile	2.9 A<br>3.1 A<br>3.9 A	1x hb to H:73:Asn	1	0	0	0	0	0.72	77.30%
A:452:Leu				0	0	0	0	0	0.38	16.80%
A:455:Leu	H:101:Tyr	3.9 A		0	0	0	0	0	0.72	38.70%
A:456:Phe	H:101:Tyr	3.7 A		0	0	0	0	0	0.77	20.70%
A:473:Tyr	H:101:Tyr	3.6 A		0	0	0	0	0	0.67	51.00%
A:475:Ala	H:105:Tyr	2.7 A	1x hb to H:105:Tyr	1	0	0	0	0	0.74	75.20%
A:476:Gly				0	0	0	0	0	0	28.80%
A:477:Ser				0	0	0	0	0	0	7.60%
A:478:Thr				0	0	0	0	0	0	0.30%
A:484:Lys				0	0	0	0	0	0	17.50%
A:485:Gly	H:52:Trp	3.9 A		0	0	0	0	0	0.78	72.10%
A:486:Phe	L:93:Trp<br>L:34:Tyr<br>H:52:Trp	3.4 A<br>3.5 A<br>4.0 A		0	0	0	0	0	0.69	85.00%
A:487:Asn	L:34:Tyr<br>H:105:Tyr	3.2 A<br>3.8 A		0	0	0	0	0	0.66	93.80%
A:489:Tyr	H:98:Lys<br>H:100:Tyr<br>H:52:Trp	3.2 A<br>3.6 A<br>3.8 A		0	0	0	0	0	0.8	97.30%
A:490:Phe				0	0	0	0	0	0.34	52.80%
A:491:Pro	H:101:Tyr	3.4 A		0	0	0	0	0	0.63	68.00%
A:492:Leu				0	0	0	0	0	0.09	27.00%
A:493:Gln	H:31:Asn<br>H:101:Tyr	2.8 A<br>3.8 A	1x hb to H:31:Asn	1	0	0	0	0	0.66	96.00%
A:494:Ser	H:31:Asn<br>H:28:Ser	2.6 A<br>3.6 A	3x hb to H:31:Asn	3	0	0	0	0	0.82	99.20%
A:496:Gly				0	0	0	0	0	0.83	89.90%
A:498:Gln				0	0	0	0	0	0.65	90.40%
A:501:Tyr	H:26:Gly<br>H:27:Phe<br>H:25:Ser	3.0 A<br>3.2 A<br>3.3 A	2x clash to H:26:Gly	0	0	0	0	2	0.77	59.20%
A:505:Tyr	H:26:Gly<br>H:27:Phe	3.4 A<br>3.6 A		0	0	0	0	0	0.78	55.10%

H:24:Val				0	0	0	0	0	0.5	16.40%
H:25:Ser	A:501:Tyr	3.3 A		0	0	0	0	0	0.64	29.10%
H:26:Gly	A:501:Tyr<br>A:505:Tyr	3.0 A<br>3.4 A	2x clash to A:501:Tyr	0	0	0	0	2	0.78	68.20%
H:27:Phe	A:501:Tyr<br>A:505:Tyr	3.2 A<br>3.6 A		0	0	0	0	0	0.71	81.90%
H:28:Ser	A:449:Tyr<br>A:494:Ser	3.1 A<br>3.6 A		0	0	0	0	0	0.78	98.50%
H:29:Leu				0	0	0	0	0	0.23	94.40%
H:30:Ile	A:449:Tyr	3.9 A		0	0	0	0	0	0.58	72.40%
H:31:Asn	A:494:Ser<br>A:493:Gln	2.6 A<br>2.8 A	1x hb to A:493:Gln 3x hb to A:494:Ser	4	0	0	0	0	0.75	91.20%
H:32:Tyr				0	0	0	0	0	0	9.70%
H:50:Val				0	0	0	0	0	0	5.50%
H:52:Trp	A:489:Tyr<br>A:485:Gly<br>A:486:Phe	3.8 A<br>3.9 A<br>4.0 A		0	0	0	0	0	0.78	69.40%
H:53:Thr				0	0	0	0	0	0.25	93.10%
H:54:Gly				0	0	0	0	0	0.23	26.30%
H:58:Asn				0	0	0	0	0	0.69	27.60%
H:73:Asn	A:449:Tyr<br>A:446:Gly	2.9 A<br>3.9 A	1x hb to A:449:Tyr	1	0	0	0	0	0.63	43.60%
H:74:Ser	A:446:Gly	3.9 A		0	0	0	0	0	0.58	25.20%
H:76:Ser				0	0	0	0	0	0.3	67.50%
H:98:Lys	A:489:Tyr	3.2 A		0	0	0	0	0	0.76	57.30%
H:99:Asp				0	0	0	0	0	0	0.20%
H:100:Tyr	A:489:Tyr	3.6 A		0	0	0	0	0	0.71	92.90%
H:101:Tyr	A:491:Pro<br>A:473:Tyr<br>A:456:Phe<br>A:493:Gln<br>A:455:Leu	3.4 A<br>3.6 A<br>3.7 A<br>3.8 A<br>3.9 A		0	0	0	0	0	0.69	76.60%
H:102:Gly				0	0	0	0	0	0	13.00%
H:105:Tyr	A:475:Ala<br>A:487:Asn	2.7 A<br>3.8 A	1x hb to A:475:Ala	1	0	0	0	0	0.57	56.00%
L:32:Ser				0	0	0	0	0	0	4.40%
L:34:Tyr	A:487:Asn<br>A:486:Phe	3.2 A<br>3.5 A		0	0	0	0	0	0.83	65.30%
L:93:Trp	A:486:Phe	3.4 A		0	0	0	0	0	0.7	46.40%
L:98:Trp				0	0	0	0	0	0.56	31.90%

Table S6. Protein-Protein Interactions for the Gamma RBD/2B04.

Residue	Closest	Distance	Specific Interactions	# HB	# Salt Bridges	# Pi Stacking	# Disulfides	# vdW Clash	Surface Complementarity	Buried SASA
B:446:Gly				0	0	0	0	0	0	19.00%
B:449:Tyr	H:26:Gly<br>H:27:Phe<br>H:28:Ser	3.4 A<br>3.6 A<br>3.8 A		0	0	0	0	0	0.79	64.90%
B:450:Asn				0	0	0	0	0	0	2.90%
B:452:Leu	H:30:Ile	3.7 A		0	0	0	0	0	0.89	46.30%
B:453:Tyr				0	0	0	0	0	0	1.80%
B:455:Leu	H:101:Tyr	3.5 A		0	0	0	0	0	0.94	18.90%
B:456:Phe				0	0	0	0	0	0.77	21.20%
B:472:Ile				0	0	0	0	0	0	6.20%
B:483:Val				0	0	0	0	0	0	3.00%
B:484:Lys	H:57:Thr<br>H:58:Asn<br>H:52:Trp	3.0 A<br>3.3 A<br>3.7 A	1x hb to H:57:Thr<br>1x hb to H:58:Asn	2	0	0	0	0	0.79	50.20%
B:485:Gly	H:52:Trp	3.9 A		0	0	0	0	0	0.8	92.80%
B:486:Phe	H:100:Tyr	4.0 A		0	0	0	0	0	0.84	43.10%
B:488:Cys				0	0	0	0	0	0	4.80%
B:489:Tyr	H:101:Tyr	3.3 A	1x pi stack to H:101:Tyr	0	0	1	0	0	0.8	76.70%
B:490:Phe	H:101:Tyr<br>H:54:Gly<br>H:53:Thr	2.8 A<br>3.4 A<br>3.7 A	1x hb, 1x clash to H:101:Tyr	1	0	0	0	1	0.76	59.30%
B:492:Leu	H:30:Ile	3.1 A		0	0	0	0	0	0.81	27.70%
B:493:Gln	H:101:Tyr<br>H:31:Asn<br>H:30:Ile	3.4 A<br>3.5 A<br>3.6 A		0	0	0	0	0	0.86	86.10%
B:494:Ser	H:28:Ser<br>H:31:Asn<br>H:30:Ile	2.6 A<br>2.7 A<br>3.5 A	1x hb to H:28:Ser<br>2x hb to H:31:Asn	3	0	0	0	0	0.8	88.10%
B:498:Gln	H:1:Gln	3.9 A		0	0	0	0	0	0	44.40%
B:501:Tyr	H:1:Gln	3.0 A	1x hb to H:1:Gln	1	0	0	0	0	0.9	25.60%
H:1:Gln	B:501:Tyr<br>B:498:Gln	3.0 A<br>3.9 A	1x hb to B:501:Tyr	1	0	0	0	0	0.87	29.50%
H:25:Ser				0	0	0	0	0	0	18.90%
H:26:Gly	B:449:Tyr	3.4 A		0	0	0	0	0	0.65	63.60%
H:27:Phe	B:449:Tyr	3.6 A		0	0	0	0	0	0.79	74.10%

H:28:Ser	B:494:Ser	2.6 A	1x hb to							
	B:449:Tyr	3.8 A	B:494:Ser	1	0	0	0	0	0.76	86.40%
H:30:Ile	B:492:Leu	3.1 A								
	B:494:Ser	3.5 A								
	B:493:Gln	3.6 A								
	B:452:Leu	3.7 A		0	0	0	0	0	0.83	76.90%
H:31:Asn	B:494:Ser	2.7 A	2x hb to							
	B:493:Gln	3.5 A	B:494:Ser	2	0	0	0	0	0.85	73.10%
H:52:Trp	B:484:Lys	3.7 A								
	B:485:Gly	3.9 A		0	0	0	0	0	0.8	59.00%
H:53:Thr	B:490:Phe	3.7 A		0	0	0	0	0	0.4	74.20%
H:54:Gly	B:490:Phe	3.4 A		0	0	0	0	0	0.87	56.20%
H:55:Gly				0	0	0	0	0	0	13.40%
H:56:Gly				0	0	0	0	0	0.78	27.40%
H:57:Thr			1x hb to							
	B:484:Lys	3.0 A	B:484:Lys	1	0	0	0	0	0.79	39.80%
H:58:Asn			1x hb to							
	B:484:Lys	3.3 A	B:484:Lys	1	0	0	0	0	0.87	39.40%
H:74:Ser				0	0	0	0	0	0	2.70%
H:76:Ser				0	0	0	0	0	0	20.10%
H:98:Lys				0	0	0	0	0	0	1.90%
H:100:Tyr	B:486:Phe	4.0 A		0	0	0	0	0	0.81	91.50%
H:101:Tyr	B:490:Phe	2.8 A	1x pi stack to							
	B:489:Tyr	3.3 A	B:489:Tyr							
	B:493:Gln	3.4 A	1x hb, 1x clash							
	B:455:Leu	3.5 A	to B:490:Phe	1	0	1	0	1	0.83	78.70%
H:102:Gly				0	0	0	0	0	0	7.00%
H:105:Tyr				0	0	0	0	0	0	38.30%
L:34:Tyr				0	0	0	0	0	0	9.10%

Table S7. Protein-Protein Interactions for the Delta RBD/2B04.

Residue	Closest	Distance	Specific Interactions	# HB	# Salt Bridges	# Pi Stacking	# Disulfides	# vdW Clash	Surface Complementarity	Buried SASA
E:446:Gly				0	0	0	0	0	0	2.50%
	H:27:Phe	3.2 A								
	H:28:Ser	3.6 A								
E:449:Tyr	H:26:Gly	3.6 A		0	0	0	0	0	0.87	65.60%
E:452:Arg				0	0	0	0	0	0.58	11.30%
E:453:Tyr				0	0	0	0	0	0	0.10%
E:455:Leu	H:101:Tyr	3.7 A		0	0	0	0	0	0.83	45.20%
E:483:Val				0	0	0	0	0	0	4.00%
	H:53:Thr	2.8 A	1x hb to							
	H:54:Gly	2.9 A	H:53:Thr							
	H:56:Gly	2.9 A	1x hb to							
	H:55:Gly	3.1 A	H:54:Gly							
	H:52:Trp	3.4 A	1x hb to							
E:484:Glu	H:58:Asn	3.5 A	H:56:Gly	3	0	0	0	0	0.76	90.50%
	H:52:Trp	3.5 A								
E:485:Gly	H:58:Asn	3.9 A		0	0	0	0	0	0.79	81.80%
			1x pi stack to							
			L:34:Tyr							
			1x pi stack to							
E:486:Phe	L:34:Tyr	3.5 A	L:93:Trp	0	0	2	0	0	0.76	55.20%
E:488:Cys				0	0	0	0	0	0.08	32.70%
	H:101:Tyr	3.4 A								
E:489:Tyr	H:100:Tyr	3.5 A		0	0	0	0	0	0.85	63.80%
E:490:Phe	H:53:Thr	3.9 A		0	0	0	0	0	0.5	55.20%
E:491:Pro				0	0	0	0	0	0.02	1.40%
E:492:Leu	H:30:Ile	3.3 A		0	0	0	0	0	0.6	77.50%
	H:31:Asn	3.3 A								
E:493:Gln	H:101:Tyr	3.4 A		0	0	0	0	0	0.82	75.60%
			1x hb to							
	H:28:Ser	2.8 A	H:28:Ser							
	H:31:Asn	2.8 A	3x hb to							
E:494:Ser	H:30:Ile	3.1 A	H:31:Asn	4	0	0	0	0	0.73	89.90%
E:495:Tyr				0	0	0	0	0	0	13.90%
E:496:Gly				0	0	0	0	0	0	4.20%
E:498:Gln				0	0	0	0	0	0	0.60%
H:25:Ser				0	0	0	0	0	0	2.60%
H:26:Gly	E:449:Tyr	3.6 A		0	0	0	0	0	0.77	27.50%
H:27:Phe	E:449:Tyr	3.2 A		0	0	0	0	0	0.77	59.20%
	E:494:Ser	2.8 A	1x hb to							
H:28:Ser	E:449:Tyr	3.6 A	E:494:Ser	1	0	0	0	0	0.84	86.00%

H:29:Leu				0	0	0	0	0	0.08	65.00%
H:30:Ile	E:494:Ser<br>E:492:Leu	3.1 A<br>3.3 A		0	0	0	0	0	0.59	81.10%
H:31:Asn	E:494:Ser<br>E:493:Gln	2.8 A<br>3.3 A	3x hb to E:494:Ser	3	0	0	0	0	0.84	78.70%
H:32:Tyr				0	0	0	0	0	0	1.90%
H:52:Trp	E:484:Glu<br>E:485:Gly	3.4 A<br>3.5 A		0	0	0	0	0	0.83	79.30%
H:53:Thr	E:484:Glu<br>E:490:Phe	2.8 A<br>3.9 A	1x hb to E:484:Glu	1	0	0	0	0	0.48	83.10%
H:54:Gly	E:484:Glu	2.9 A	1x hb to E:484:Glu	1	0	0	0	0	0.65	44.40%
H:55:Gly	E:484:Glu	3.1 A		0	0	0	0	0	0.79	0.00%
H:56:Gly	E:484:Glu	2.9 A	1x hb to E:484:Glu	1	0	0	0	0	0.82	48.90%
H:58:Asn	E:484:Glu<br>E:485:Gly	3.5 A<br>3.9 A		0	0	0	0	0	0.86	24.90%
H:73:Asn				0	0	0	0	0	0	7.50%
H:76:Ser				0	0	0	0	0	0.01	25.70%
H:98:Lys				0	0	0	0	0	0	9.10%
H:99:Asp				0	0	0	0	0	0	0.10%
H:100:Tyr	E:489:Tyr	3.5 A		0	0	0	0	0	0.21	64.10%
H:101:Tyr	E:493:Gln<br>E:489:Tyr<br>E:455:Leu	3.4 A<br>3.4 A<br>3.7 A		0	0	0	0	0	0.84	40.80%
H:105:Tyr				0	0	0	0	0	0	3.50%
L:34:Tyr	E:486:Phe	3.5 A	1x pi stack to E:486:Phe	0	0	1	0	0	0.77	24.80%
L:93:Trp			1x pi stack to E:486:Phe	0	0	1	0	0	0	49.70%
L:96:Asn				0	0	0	0	0	0	1.30%
L:98:Trp				0	0	0	0	0	0	8.30%

Table S8. Protein-Protein Interactions for the Kappa RBD/2B04.

Residue	Closest	Distance	Specific Interactions	# HB	# Salt Bridges	# Pi Stacking	# Disulfides	# vdW Clash	Surface Complementarity	Buried SASA
A:446:Gly				0	0	0	0	0	0	22.40%
A:449:Tyr	H:28:Ser<br>H:31:Asn<br>H:27:Phe	3.4 A<br>3.7 A<br>3.9 A		0	0	0	0	0	0.92	65.30%
A:452:Arg				0	0	0	0	0	0.5	14.10%
A:455:Leu	H:101:Tyr	3.9 A		0	0	0	0	0	0.73	50.40%
A:456:Phe				0	0	0	0	0	0.5	9.30%
A:472:Ile				0	0	0	0	0	0	5.50%
A:482:Gly				0	0	0	0	0	0	0.20%
A:483:Val	H:58:Asn	3.8 A		0	0	0	0	0	0.85	17.90%
A:484:Gln	H:53:Thr<br>H:52:Trp<br>H:58:Asn<br>H:54:Gly<br>H:100:Tyr<br>H:101:Tyr	3.0 A<br>3.0 A<br>3.1 A<br>3.2 A<br>3.5 A<br>3.7 A	1x clash to H:52:Trp<br>1x hb to H:53:Thr<br>1x hb to H:54:Gly<br>1x hb to H:58:Asn	3	0	0	0	1	0.81	97.60%
A:485:Gly	H:100:Tyr<br>H:52:Trp	2.7 A<br>3.9 A	1x hb to H:100:Tyr	1	0	0	0	0	0.63	78.70%
A:486:Phe	L:34:Tyr<br>H:105:Tyr	3.2 A<br>3.4 A	1x pi stack to H:105:Tyr	0	0	1	0	0	0.85	71.60%
A:487:Asn				0	0	0	0	0	0.6	6.10%
A:488:Cys	H:100:Tyr	3.0 A		0	0	0	0	0	0.74	0.00%
A:489:Tyr	H:101:Tyr<br>H:100:Tyr	3.6 A<br>3.7 A		0	0	0	0	0	0.69	63.90%
A:490:Phe	H:101:Tyr<br>H:54:Gly<br>H:53:Thr	2.7 A<br>3.5 A<br>3.5 A	1x hb to H:101:Tyr	1	0	0	0	0	0.82	66.80%
A:492:Leu	H:30:Ile	3.9 A		0	0	0	0	0	0.52	78.70%
A:493:Gln	H:31:Asn<br>H:30:Ile	3.6 A<br>3.9 A		0	0	0	0	0	0.73	50.40%
A:494:Ser	H:31:Asn<br>H:28:Ser<br>H:30:Ile	2.9 A<br>3.0 A<br>3.8 A	1x clash to H:28:Ser<br>3x hb to H:31:Asn	3	0	0	0	1	0.84	92.50%
A:498:Gln				0	0	0	0	0	0	21.40%
H:1:Gln				0	0	0	0	0	0	5.50%
H:25:Ser				0	0	0	0	0	0	6.90%
H:26:Gly				0	0	0	0	0	0	52.10%

H:27:Phe	A:449:Tyr	3.9 A		0	0	0	0	0	0.62	65.30%
H:28:Ser	A:494:Ser	3.0 A	1x clash to							
	A:449:Tyr	3.4 A	A:494:Ser	0	0	0	0	1	0.89	81.30%
H:30:Ile	A:494:Ser	3.8 A								
	A:493:Gln	3.9 A								
	A:492:Leu	3.9 A		0	0	0	0	0	0.78	79.80%
H:31:Asn	A:494:Ser	2.9 A								
	A:493:Gln	3.6 A	3x hb to							
	A:449:Tyr	3.7 A	A:494:Ser	3	0	0	0	0	0.84	76.00%
H:52:Trp	A:484:Gln	3.0 A	1x clash to							
	A:485:Gly	3.9 A	A:484:Gln	0	0	0	0	1	0.81	79.00%
H:53:Thr	A:484:Gln	3.0 A	1x hb to							
	A:490:Phe	3.5 A	A:484:Gln	1	0	0	0	0	0.81	85.20%
H:54:Gly	A:484:Gln	3.2 A	1x hb to							
	A:490:Phe	3.5 A	A:484:Gln	1	0	0	0	0	0.93	91.10%
H:55:Gly				0	0	0	0	0	0	3.80%
H:56:Gly				0	0	0	0	0	0	21.00%
H:57:Thr				0	0	0	0	0	0	3.50%
H:58:Asn	A:484:Gln	3.1 A	1x hb to							
	A:483:Val	3.8 A	A:484:Gln	1	0	0	0	0	0.83	51.70%
H:98:Lys				0	0	0	0	0	0.18	41.60%
H:100:Tyr	A:485:Gly	2.7 A								
	A:488:Cys	3.0 A								
	A:484:Gln	3.5 A	1x hb to							
	A:489:Tyr	3.7 A	A:485:Gly	1	0	0	0	0	0.7	96.20%
H:101:Tyr	A:490:Phe	2.7 A								
	A:489:Tyr	3.6 A								
	A:484:Gln	3.7 A	1x hb to							
	A:455:Leu	3.9 A	A:490:Phe	1	0	0	0	0	0.79	65.10%
H:105:Tyr			1x pi stack to							
	A:486:Phe	3.4 A	A:486:Phe	0	0	1	0	0	0.8	26.20%
L:34:Tyr	A:486:Phe	3.2 A		0	0	0	0	0	0.83	43.30%
L:93:Trp				0	0	0	0	0	0.27	12.10%
L:98:Trp				0	0	0	0	0	0.6	47.10%

Table S9. Protein-Protein Interactions for the Epsilon RBD/2B04.

Residue	Closest	Distance	Specific Interactions	# HB	# Salt Bridges	# Pi Stacking	# Disulfides	# vdW Clash	Surface Complementarity	Buried SASA
A:444:Lys	H:74:Ser	3.5 A		0	0	0	0	0	0.91	16.60%
A:445:Val				0	0	0	0	0	0	0.60%
A:446:Gly	H:74:Ser	3.4 A		0	0	0	0	0	0.92	42.60%
A:447:Gly	H:74:Ser	3.6 A		0	0	0	0	0	0.88	100.00%
A:449:Tyr	H:73:Asn	3.8 A		0	0	0	0	0	0.75	57.60%
A:452:Arg	H:54:Gly	3.5 A		0	0	0	0	0	0	17.90%
A:483:Val	H:58:Asn	3.4 A		0	0	0	0	0	0.82	57.20%
A:484:Glu	H:100:Tyr<br>H:54:Gly<br>H:52:Trp<br>H:53:Thr	2.8 A<br>3.0 A<br>3.4 A<br>3.5 A	1x hb to H:54:Gly<br>1x hb to H:100:Tyr	2	0	0	0	0	0.82	92.30%
A:485:Gly	H:100:Tyr	3.2 A		0	0	0	0	0	0.82	71.70%
A:486:Phe	H:100:Tyr<br>H:105:Tyr<br>H:102:Gly<br>H:104:Tyr	2.9 A<br>3.4 A<br>3.7 A<br>3.9 A		0	0	0	0	0	0.84	71.40%
A:487:Asn				0	0	0	0	0	0	1.00%
A:488:Cys				0	0	0	0	0	0.87	0.00%
A:489:Tyr	H:101:Tyr	3.6 A		0	0	0	0	0	0.75	57.10%
A:490:Phe	H:54:Gly	3.5 A		0	0	0	0	0	0.78	52.00%
A:492:Leu				0	0	0	0	0	0	10.90%
A:493:Gln				0	0	0	0	0	0	20.40%
A:494:Ser				0	0	0	0	0	0.71	46.90%
H:30:Ile				0	0	0	0	0	0.78	52.00%
H:31:Asn				0	0	0	0	0	0	14.80%
H:52:Trp	A:484:Glu	3.4 A		0	0	0	0	0	0.78	68.20%
H:53:Thr	A:484:Glu	3.5 A		0	0	0	0	0	0.92	48.70%
H:54:Gly	A:484:Glu<br>A:490:Phe<br>A:452:Arg	3.0 A<br>3.5 A<br>3.5 A	1x hb to A:484:Glu	1	0	0	0	0	0.72	81.60%
H:56:Gly				0	0	0	0	0	0	14.80%
H:57:Thr				0	0	0	0	0	0.23	17.30%
H:58:Asn	A:483:Val	3.4 A		0	0	0	0	0	0.75	38.60%
H:72:Asp				0	0	0	0	0	0	2.30%
H:73:Asn	A:449:Tyr	3.8 A		0	0	0	0	0	0.77	40.70%
H:74:Ser	A:446:Gly<br>A:444:Lys<br>A:447:Gly	3.4 A<br>3.5 A<br>3.6 A		0	0	0	0	0	0.87	83.50%

H:75:Lys				0	0	0	0	0	0	0.60%
H:98:Lys				0	0	0	0	0	0.46	30.30%
H:99:Asp				0	0	0	0	0	0.45	0.20%
H:100:Tyr	A:484:Glu<br>A:486:Phe<br>A:485:Gly	2.8 A<br>2.9 A<br>3.2 A	1x hb to<br>A:484:Glu	1	0	0	0	0	0.83	98.90%
H:101:Tyr	A:489:Tyr	3.6 A		0	0	0	0	0	0.62	34.80%
H:102:Gly	A:486:Phe	3.7 A		0	0	0	0	0	0.81	4.90%
H:103:Arg				0	0	0	0	0	0.68	3.20%
H:104:Tyr	A:486:Phe	3.9 A		0	0	0	0	0	0.83	6.90%
H:105:Tyr	A:486:Phe	3.4 A		0	0	0	0	0	0.9	48.00%
L:34:Tyr				0	0	0	0	0	0	1.20%

Table S10. Protein-Protein Interactions for the Epsilon* RBD/2B04.

Residue	Closest	Distance	Specific Interactions	# HB	# Salt Bridges	# Pi Stacking	# Disulfides	# vdW Clash	Surface Complementarity	Buried SASA
A:417:Lys				0	0	0	0	0	0	9.70%
A:449:Tyr	H:28:Ser<br>H:30:Ile	3.5 A<br>3.9 A		0	0	0	0	0	0.78	50.30%
A:452:Arg				0	0	0	0	0	0	2.10%
A:455:Leu	H:101:Tyr	3.8 A		0	0	0	0	0	0.83	60.70%
A:456:Phe	H:101:Tyr	3.9 A		0	0	0	0	0	0.73	40.60%
A:483:Val				0	0	0	0	0	0	2.80%
A:484:Glu	H:54:Gly<br>H:100:Tyr<br>H:53:Thr<br>H:52:Trp	2.8 A<br>3.3 A<br>3.4 A<br>4.0 A	1x hb to<br>H:54:Gly	1	0	0	0	0	0.56	85.50%
A:485:Gly	H:52:Trp	3.8 A		0	0	0	0	0	0.57	61.60%
A:486:Phe	H:58:Asn<br>L:93:Trp<br>H:52:Trp	3.3 A<br>3.6 A<br>3.7 A	1x pi stack to<br>L:93:Trp	0	0	1	0	0	0.86	64.50%
A:489:Tyr	H:100:Tyr	3.4 A		0	0	0	0	0	0.67	71.70%
A:490:Phe	H:101:Tyr	2.5 A	1x hb to<br>H:101:Tyr	1	0	0	0	0	0.71	21.10%
A:491:Pro				0	0	0	0	0	0.88	19.20%
A:492:Leu	H:31:Asn<br>H:101:Tyr	2.6 A<br>3.5 A	1x hb to<br>H:31:Asn	1	0	0	0	0	0.8	99.60%
A:493:Gln	H:31:Asn<br>H:101:Tyr	3.0 A<br>3.7 A		0	0	0	0	0	0.72	64.30%
A:494:Ser	H:31:Asn<br>H:30:Ile	3.2 A<br>3.6 A	1x hb to<br>H:31:Asn	1	0	0	0	0	0.92	66.90%
A:498:Gln				0	0	0	0	0	0	12.90%
H:26:Gly				0	0	0	0	0	0	13.20%
H:27:Phe				0	0	0	0	0	0	29.00%
H:28:Ser	A:449:Tyr	3.5 A		0	0	0	0	0	0.83	63.50%
H:30:Ile	A:494:Ser<br>A:449:Tyr	3.6 A<br>3.9 A		0	0	0	0	0	0.82	47.30%
H:31:Asn	A:492:Leu<br>A:493:Gln<br>A:494:Ser	2.6 A<br>3.0 A<br>3.2 A	1x hb to<br>A:492:Leu<br>1x hb to<br>A:494:Ser	2	0	0	0	0	0.77	89.20%
H:32:Tyr				0	0	0	0	0	0	2.80%
H:52:Trp	A:486:Phe<br>A:485:Gly<br>A:484:Glu	3.7 A<br>3.8 A<br>4.0 A		0	0	0	0	0	0.62	86.70%
H:53:Thr	A:484:Glu	3.4 A		0	0	0	0	0	0	17.20%

H:54:Gly			1x hb to							
	A:484:Glu	2.8 A	A:484:Glu	1	0	0	0	0	0.56	25.20%
H:56:Gly				0	0	0	0	0	0.48	46.80%
H:57:Thr				0	0	0	0	0	0	0.20%
H:58:Asn	A:486:Phe	3.3 A		0	0	0	0	0	0.89	39.10%
H:99:Asp				0	0	0	0	0	0	13.50%
H:100:Tyr	A:484:Glu	3.3 A								
	A:489:Tyr	3.4 A		0	0	0	0	0	0.7	80.20%
H:101:Tyr	A:490:Phe	2.5 A								
	A:492:Leu	3.5 A								
	A:493:Gln	3.7 A								
	A:455:Leu	3.8 A	1x hb to							
	A:456:Phe	3.9 A	A:490:Phe	1	0	0	0	0	0.77	91.20%
H:102:Gly				0	0	0	0	0	0	1.60%
H:103:Arg				0	0	0	0	0	0	5.10%
L:93:Trp			1x pi stack to							
	A:486:Phe	3.6 A	A:486:Phe	0	0	1	0	0	0.91	59.60%
L:95:Asn				0	0	0	0	0	0	4.60%
L:96:Asn				0	0	0	0	0	0	0.50%

Table S11. Protein-Protein Interactions for the Omicron BA.1 RBD/2B04.

Residue	Closest	Distance	Specific Interactions	# HB	# Salt Bridges	# Pi Stacking	# Disulfides	# vdW Clash	Surface Complementarity	Buried SASA
A:446:Ser				0	0	0	0	0	0	1.30%
A:449:Tyr	H:27:Phe<br>H:26:Gly<br>H:28:Ser	3.2 A<br>3.5 A<br>3.7 A		0	0	0	0	0	0.78	63.40%
A:452:Leu				0	0	0	0	0	0.84	34.80%
A:455:Leu	H:101:Tyr	4.0 A		0	0	0	0	0	0	32.60%
A:472:Ile				0	0	0	0	0	0	3.00%
A:483:Val	H:55:Gly<br>H:56:Gly	3.6 A<br>4.0 A		0	0	0	0	0	0.85	36.30%
A:484:Ala	H:55:Gly<br>H:52:Trp<br>H:53:Thr	2.9 A<br>3.4 A<br>3.7 A	1x hb to H:55:Gly	1	0	0	0	0	0.83	91.60%
A:485:Gly	H:52:Trp<br>H:100:Tyr	3.7 A<br>3.7 A		0	0	0	0	0	0.61	86.30%
A:486:Phe				0	0	0	0	0	0.38	16.40%
A:487:Asn				0	0	0	0	0	0	1.60%
A:488:Cys	H:100:Tyr	3.9 A		0	0	0	0	0	0.87	2.90%
A:489:Tyr	H:101:Tyr<br>H:100:Tyr	3.4 A<br>3.7 A	1x pi stack to H:100:Tyr	0	0	1	0	0	0.8	61.80%
A:490:Phe	H:100:Tyr<br>H:54:Gly<br>H:53:Thr	2.9 A<br>3.5 A<br>3.8 A	1x hb to H:100:Tyr	1	0	0	0	0	0.8	68.00%
A:491:Pro				0	0	0	0	0	0	4.10%
A:492:Leu				0	0	0	0	0	0.7	39.90%
A:493:Arg	H:31:Asn	3.0 A		0	0	0	0	0	0.77	56.10%
A:494:Ser	H:28:Ser<br>H:31:Asn<br>H:30:Ile	2.6 A<br>2.8 A<br>3.9 A	1x hb to H:28:Ser<br>3x hb to H:31:Asn	4	0	0	0	0	0.7	97.70%
A:495:Tyr				0	0	0	0	0	0	6.80%
A:496:Ser				0	0	0	0	0	0	20.20%
A:498:Arg	H:1:Gln	3.5 A		0	0	0	0	0	0.49	60.60%
H:1:Gln	A:498:Arg	3.5 A		0	0	0	0	0	0.78	22.10%
H:25:Ser				0	0	0	0	0	0	4.30%
H:26:Gly	A:449:Tyr	3.5 A		0	0	0	0	0	0.66	81.50%
H:27:Phe	A:449:Tyr	3.2 A		0	0	0	0	0	0.84	78.30%
H:28:Ser	A:494:Ser<br>A:449:Tyr	2.6 A<br>3.7 A	1x hb to A:494:Ser	1	0	0	0	0	0.75	90.70%
H:30:Ile	A:494:Ser	3.9 A		0	0	0	0	0	0.7	76.20%

H:31:Asn	A:494:Ser	2.8 A	3x hb to							
	A:493:Arg	3.0 A	A:494:Ser	3	0	0	0	0	0.78	93.10%
H:32:Tyr				0	0	0	0	0	0	14.70%
H:52:Trp	A:484:Ala	3.4 A								
	A:485:Gly	3.7 A		0	0	0	0	0	0.75	65.10%
H:53:Thr	A:484:Ala	3.7 A								
	A:490:Phe	3.8 A		0	0	0	0	0	0.84	74.80%
H:54:Gly	A:490:Phe	3.5 A		0	0	0	0	0	0.82	80.80%
H:55:Gly	A:484:Ala	2.9 A	1x hb to							
	A:483:Val	3.6 A	A:484:Ala	1	0	0	0	0	0.73	47.50%
H:56:Gly	A:483:Val	4.0 A		0	0	0	0	0	0.79	51.20%
H:57:Thr				0	0	0	0	0	0	3.10%
H:58:Asn				0	0	0	0	0	0	9.40%
H:76:Ser				0	0	0	0	0	0	13.60%
H:99:Asp				0	0	0	0	0	0	5.80%
H:100:Tyr	A:490:Phe	2.9 A	1x pi stack to							
	A:489:Tyr	3.7 A	A:489:Tyr							
	A:485:Gly	3.7 A	1x hb to							
	A:488:Cys	3.9 A	A:490:Phe	1	0	1	0	0	0.77	72.80%
H:101:Tyr	A:489:Tyr	3.4 A								
	A:455:Leu	4.0 A		0	0	0	0	0	0.82	48.80%
L:34:Tyr				0	0	0	0	0	0	9.40%
L:93:Trp				0	0	0	0	0	0	6.90%

Table S12. Protein-Protein Interactions for the Omicron JN.1 RBD/2B04.

Residue	Closest	Distance	Specific Interactions	# HB	# Salt Bridges	# Pi Stacking	# Disulfides	# vdW Clash	Surface Complementarity	Buried SASA
A:445:His				0	0	0	0	0	0	3.00%
A:446:Ser				0	0	0	0	0	0	20.40%
A:449:Tyr	H:28:Ser	3.6 A								
	H:27:Phe	3.7 A		0	0	0	0	0	0.73	73.40%
A:452:Trp	H:30:Ile	4.0 A		0	0	0	0	0	0.66	34.00%
A:453:Tyr				0	0	0	0	0	0	0.70%
A:482:Gly				0	0	0	0	0	0	0.50%
A:483:Val	H:58:Asn	3.6 A		0	0	0	0	0	0.33	25.80%
A:484:Lys	H:55:Gly	3.0 A								
	H:52:Trp	3.2 A								
	H:54:Gly	3.7 A								
	H:100:Tyr	3.7 A	1x hb to							
	H:53:Thr	3.9 A	H:55:Gly	1	0	0	0	0	0.86	85.70%
A:485:Gly	H:52:Trp	3.7 A								
	H:100:Tyr	3.8 A		0	0	0	0	0	0.77	82.90%
A:486:Pro				0	0	0	0	0	0	11.70%
A:488:Cys				0	0	0	0	0	0.93	0.00%
A:489:Tyr	H:101:Tyr	3.9 A		0	0	0	0	0	0.82	53.30%
A:490:Phe	H:53:Thr	3.3 A								
	H:54:Gly	3.4 A								
	H:101:Tyr	3.5 A								
	H:100:Tyr	3.9 A		0	0	0	0	0	0.86	87.30%
A:492:Leu	H:30:Ile	3.9 A		0	0	0	0	0	0.32	98.60%
A:493:Gln	H:31:Asn	3.3 A								
	H:101:Tyr	3.8 A		0	0	0	0	0	0.65	65.90%
A:494:Ser			1x clash to							
	H:28:Ser	2.7 A	H:28:Ser							
	H:31:Asn	2.7 A	3x hb, 1x clash							
	H:30:Ile	3.7 A	to H:31:Asn	3	0	0	0	2	0.75	90.90%
A:498:Arg	H:26:Gly	3.4 A								
	H:1:Gln	3.9 A		0	0	0	0	0	0.64	61.50%
A:501:Tyr				0	0	0	0	0	0	1.50%
H:1:Gln	A:498:Arg	3.9 A		0	0	0	0	0	0	16.20%
H:24:Val				0	0	0	0	0	0	28.60%
H:25:Ser				0	0	0	0	0	0.29	33.90%
H:26:Gly	A:498:Arg	3.4 A		0	0	0	0	0	0.55	69.70%
H:27:Phe	A:449:Tyr	3.7 A		0	0	0	0	0	0.66	64.30%
H:28:Ser	A:494:Ser	2.7 A	1x clash to							
	A:449:Tyr	3.6 A	A:494:Ser	0	0	0	0	1	0.73	97.00%
H:29:Leu				0	0	0	0	0	0	21.90%

H:30:Ile	A:494:Ser	3.7 A								
	A:492:Leu	3.9 A								
	A:452:Trp	4.0 A		0	0	0	0	0	0.73	91.10%
H:31:Asn	A:494:Ser	2.7 A	3x hb, 1x clash							
	A:493:Gln	3.3 A	to A:494:Ser	3	0	0	0	1	0.7	74.60%
H:52:Trp	A:484:Lys	3.2 A								
	A:485:Gly	3.7 A		0	0	0	0	0	0.81	68.40%
H:53:Thr	A:490:Phe	3.3 A								
	A:484:Lys	3.9 A		0	0	0	0	0	0.94	100.00%
H:54:Gly	A:490:Phe	3.4 A								
	A:484:Lys	3.7 A		0	0	0	0	0	0.94	55.40%
H:55:Gly			1x hb to							
	A:484:Lys	3.0 A	A:484:Lys	1	0	0	0	0	0.82	24.90%
H:56:Gly				0	0	0	0	0	0	20.30%
H:58:Asn	A:483:Val	3.6 A		0	0	0	0	0	0.4	40.30%
H:73:Asn				0	0	0	0	0	0	4.50%
H:76:Ser				0	0	0	0	0	0	34.40%
H:100:Tyr	A:484:Lys	3.7 A								
	A:485:Gly	3.8 A								
	A:490:Phe	3.9 A		0	0	0	0	0	0.85	72.90%
H:101:Tyr	A:490:Phe	3.5 A								
	A:493:Gln	3.8 A								
	A:489:Tyr	3.9 A		0	0	0	0	0	0.73	58.50%
L:93:Trp				0	0	0	0	0	0	10.70%

Table S13. Results of Protein-Protein Docking.

Variant/ Cluster	Default			Masked Non-CDR		
**Wild Type**	**PIPER pose energy**	**PIPER pose score**	**RMSD**	**PIPER pose energy**	**PIPER pose score**	**RMSD**
Pose 1	**-272.051**	**-457.198**	**7.652**	-269.056	-293.516	27.802
Pose 2	-270.656	-293.516	27.802	-252.377	-440.06	6.424
Pose 3	-263.193	-388.143	12.793	-281.287	-359.293	24.796
Pose 4	-282.056	-266.848	25.443	**-264.044**	**-209.244**	**3.636**
Pose 5	-313.066	-258.313	30.705	-307.552	-349.946	29.796
Pose 6	-293.715	-387.458	31.699	-252.952	-267.746	30.367
Pose 7	-291.509	-250.088	20.062	-254.457	-406.003	30.81
Pose 8	-300.31	-250.482	33.924	-268.177	-175.706	24.518
Pose 9	-294.14	-210.379	23.62	-259.918	-371.484	14.703
Pose 10	-293.776	-401.988	31.135	-271.934	-302.371	32.476
Pose 11	-280.319	-207.697	26.147	-274.136	-309.833	18.836
Pose 12	-356.005	-221.059	40.203	-261.556	-222.226	30.728
Pose 13	-283.564	-271.411	31.654	-264.5	-206.757	23.772
Pose 14	-264.17	-322.635	32.043	-270.178	-219.762	27.687
Pose 15	-263.051	-142.35	21.821	-278.925	-339.772	26.714
Pose 16	-272.601	-317.152	14.155	-277.91	-250.482	33.924
Pose 17	-273.778	-219.762	27.687	-258.206	-251.206	27.097

Pose 18	-266.614	-142.251	30.865	-259.797	-212.289	30.731
Pose 19	-301.886	-309.501	10.305	-273.422	-247.819	30.86
Pose 20	-280.618	-335.719	30.75	-255.313	-260.417	30.501
Pose 21	-296.836	-260.176	20.946	-271.755	-263.305	24.596
Pose 22	-263.212	-226.819	13.647	-261.977	-235.555	21.586
Pose 23	-274.599	-223.198	23.631	-256.566	-264.263	21.079
Pose 24	-263.171	-206.177	22.626	-293.189	-366.239	11.629
Pose 25	-295.05	-169.366	33.64	-284.141	-267.54	24.35
Pose 26	-283.475	-215.471	23.285	-266.574	-268.67	30.326
Pose 27	-266.764	-173.55	20.517	-291.08	-281.905	21.843
Pose 28	-266.892	-187.569	28.364	-259.892	-187.569	28.364
Pose 29	-268.096	-231.283	28.364	-265.348	-293.907	27.358
Pose 30	-266.786	-195.066	32.923	-259.486	-278.004	29.732
**Alpha**	**PIPER pose energy**	**PIPER pose score**	**RMSD**	**PIPER pose energy**	**PIPER pose score**	**RMSD**
Pose 1	-296.927	-291.689	30.566	-269.386	-348.523	43.91
Pose 2	-270.586	-348.523	43.91	-272.527	-291.689	30.566
Pose 3	-276.367	-198.932	16.549	-262.74	-302.226	31.909
Pose 4	-361.976	-303.029	33.843	-259.778	-312.116	30.599
Pose 5	-287.244	-170.5	26.876	-339.376	-303.029	33.843
Pose 6	-293.751	-210.095	26.523	-293.89	-312.295	30.918
Pose 7	-296.699	-357.453	29.628	-282.88	-256.422	30.309
Pose 8	-291.376	-375.782	32.521	-283.219	-285.979	33.382
Pose 9	-291.102	-190.773	31.792	-265.403	-334.036	31.313
Pose 10	-293.89	-312.295	30.918	-272.182	-235.037	29.829
Pose 11	-285.648	-177.919	30.507	-325.225	-315.995	27.267
Pose 12	-278.588	-275.184	30.347	-280.933	-453.935	16.406
Pose 13	-284.204	-182.756	26.438	**-259.291**	**-393.827**	**12.128**
Pose 14	**-283.941**	**-402.523**	**8.721**	-261.667	-333.349	25.338
Pose 15	-275.806	-232.716	30.644	-312.971	-293.118	19.43
Pose 16	-277.584	-247.569	20.203	-263.751	-312.242	31.151
Pose 17	-270.383	-349.124	30.878	-262.386	-366.058	39.906
Pose 18	-278.452	-340.94	40.305	-273.171	-212.119	18.861
Pose 19	-272.382	-235.037	29.829	-261.816	-298.269	27.253
Pose 20	-282.847	-330.987	30.498	-265.381	-220.82	31.053
Pose 21	-269.594	-262.631	31.735	-274.869	-326.557	32.203
Pose 22	-283.18	-236.532	34.624	-272.148	-255.829	26.596
Pose 23	-316.235	-253.778	28.886	-313.035	-253.778	28.886
Pose 24	-301.168	-102.291	29.925	-269.09	-265.926	32.457
Pose 25	-274.869	-326.557	32.203	-261.659	-190.958	31.522
Pose 26	-307.497	-205.954	21.525	-272.784	-213.663	30.229
Pose 27	-273.616	-406.346	16.468	-294.263	-146.42	29.34
Pose 28	-280.634	-143.077	20.739	-268.276	-242.919	14.834
Pose 29	-278.806	-139.562	31.517	-270.816	-290.019	15.702
Pose 30	-270.838	-199.706	41.134	-262.834	-160.702	34.932
**Beta**	**PIPER pose energy**	**PIPER pose score**	**RMSD**	**PIPER pose energy**	**PIPER pose score**	**RMSD**

Pose 1	-294.107	-350.493	17.704	-272.664	-355.222	18.286
Pose 2	-255.32	-399.685	38.38	-255.725	-332.692	37.636
Pose 3	-315.257	-364.965	25.665	-255.277	-369.369	19.542
Pose 4	-281.181	-323.586	27.406	-275.981	-323.586	27.406
Pose 5	-306.162	-298.34	22.467	-251.997	-295.893	21.895
Pose 6	-257.689	-298.406	31.644	-248.909	-244.276	22.058
Pose 7	-255.597	-295.893	21.895	-255.29	-298.406	31.644
Pose 8	-257.487	-255.37	38.773	-261.058	-328.706	28.638
Pose 9	-270.701	-249.031	31.411	-247.489	-241.074	29.33
Pose 10	-257.072	-358.486	21.759	-263.547	-276.475	32.76
Pose 11	-258.365	-223.202	30.509	-249.04	-270.371	31.503
Pose 12	-268.037	-188.616	38.7	-248.095	-304.657	30.208
Pose 13	-255.274	-298.298	30.94	-251.997	-257.451	30.336
Pose 14	-284.178	-206.666	32.989	-247.142	-250.403	30.18
Pose 15	-305.68	-282.348	27.851	-267.087	-363.78	25.872
Pose 16	-282.276	-199.333	23.61	-253.177	-341.401	28.881
Pose 17	-295.101	-282.296	30.813	-265.08	-190.597	32.055
Pose 18	-281.818	-283.151	28.923	**-247.579**	**-328.352**	**13.699**
Pose 19	-255.978	-266.434	38.03	-257.271	-319.371	17.293
Pose 20	-258.248	-220.655	31.266	-259.251	-258.094	22.68
Pose 21	-257.419	-207.932	31.759	-265.275	-381.8	32.303
Pose 22	-260.19	-211.408	33.455	-248.527	-321.118	24.774
Pose 23	-266.48	-190.597	32.055	-255.561	-398.355	36.878
Pose 24	-258.818	-183.406	30.9	-245.454	-297.764	41.794
Pose 25	-301.8	-254.263	25.937	-280.864	-201.937	31.509
Pose 26	-258.68	-330.43	28.98	-246.141	-386.792	42.194
Pose 27	-272.198	-321.982	31	-297.565	-223.747	30.907
Pose 28	-260.18	-188.693	29.694	-251.986	-305.801	26.542
Pose 29	-256.18	-249.135	27.904	-256.714	-227.255	26.665
Pose 30	**-264.837**	**-139.318**	**16.436**	-260.237	-139.318	16.436
**Gamma**	**PIPER pose energy**	**PIPER pose score**	**RMSD**	**PIPER pose energy**	**PIPER pose score**	**RMSD**
Pose 1	-298.264	-370.92	27.444	-292.736	-328.16	27.688
Pose 2	-275.04	-397.502	31.51	-273.04	-397.502	31.51
Pose 3	-311.98	-345.838	31.877	-254.29	-234.478	27.433
Pose 4	-259.927	-280.101	30.808	-291.282	-341.34	30.397
Pose 5	-254.472	-343.907	28.106	-250.857	-302.214	31.962
Pose 6	-300.285	-284.464	32.164	-245.493	-233.589	32.222
Pose 7	-277.642	-245.088	32.247	-308.239	-208.745	31.609
Pose 8	-358.483	-245.035	33.18	-245.709	-385.937	13.916
Pose 9	-277.028	-121.814	24.837	-246.571	-219.276	29.92
Pose 10	-310.814	-221.709	26.847	-261.012	-234.508	26.67
Pose 11	**-295.785**	**-418.261**	**16.55**	-245.595	-273.203	27.232
Pose 12	-263.904	-297.13	36.406	-253.39	-351.028	22.352
Pose 13	-258.498	-196.152	30.024	-248.043	-342.889	26.25
Pose 14	-265.724	-198.165	31.81	-260.947	-206.06	30.662
Pose 15	-262.041	-268.36	30.757	-246.474	-317.823	30.027

Pose 16	-266.338	-268.95	29.492	-313.483	-245.035	33.18
Pose 17	-261.39	-351.028	22.352	-302.517	-421.859	19.773
Pose 18	-260.456	-179.894	28.468	-249.145	-294.143	28.335
Pose 19	-267.178	-280.655	26.668	-257.996	-276.102	28.63
Pose 20	-271.134	-171.553	28.365	-247.633	-326.279	30.488
Pose 21	-296.565	-115.94	30.4	-259.456	-179.894	28.468
Pose 22	-272.571	-59.687	27.574	-250.054	-300.824	25.796
Pose 23	-266.241	-235.979	28.984	**-252.902**	**-337.64**	**11.192**
Pose 24	-294.222	-242.167	34.134	-255.909	-259.788	27.068
Pose 25	-256.778	-188.456	25.39	-248.823	-301.358	32.816
Pose 26	-302.517	-421.859	19.773	-250.618	-288.021	25.847
Pose 27	-275.741	-204.851	25.688	-251.818	-282.357	30.121
Pose 28	-255.783	-247.258	27.357	-262.146	-245.791	26.039
Pose 29	-271.834	-150.367	30.385	-249.982	-326.368	25.733
Pose 30	-260.664	-86.034	34.573	-245.853	-332.572	28.336
**Delta**	**PIPER pose energy**	**PIPER pose score**	**RMSD**	**PIPER pose energy**	**PIPER pose score**	**RMSD**
Pose 1	-315.147	-315.786	31.474	-262.689	-257.985	31.75
Pose 2	-314.193	-310.164	31.633	-314.194	-310.164	31.633
Pose 3	-273.111	-263.401	31.635	-261.911	-263.401	31.635
Pose 4	-260.67	-374.617	29.445	-253.27	-374.617	29.445
Pose 5	-259.837	-217.793	31.312	-258.235	-305.078	27.426
Pose 6	-260.635	-305.078	27.426	-250.957	-211.293	27.629
Pose 7	-294.753	-272.962	27.326	-265.158	-263.86	27.194
Pose 8	-263.067	-234.85	32.855	-252.904	-314.253	31.352
Pose 9	-258.512	-314.827	28.62	-282.323	-290.786	32.38
Pose 10	-290.135	-289.331	24.126	-271.267	-352.679	24.038
Pose 11	-271.867	-352.679	24.038	-255.703	-253.111	31.258
Pose 12	-259.695	-308.939	31.621	-251.759	-238.022	30.191
Pose 13	-257.697	-146.105	29.834	-270.08	-197.994	29.14
Pose 14	-311.054	-148.021	34.055	-281.811	-312.967	24.262
Pose 15	-274.676	-182.6	22.423	-260.726	-304.588	32.127
Pose 16	-257.937	-134.001	30.613	-251.48	-227.697	30.598
Pose 17	-259.05	-254.217	30.466	-312.236	-308.46	30.765
Pose 18	-267.315	-319.744	30.772	-265.307	-208.603	16.924
Pose 19	-272.574	-301.979	31.271	-250.389	-298.185	31.055
Pose 20	-258.159	-247.471	30.392	-268.845	-304.268	29.487
Pose 21	-274.008	-184.593	19.847	-260.44	-389.76	36.368
Pose 22	-258.07	-276.288	29.237	-258.45	-254.217	30.466
Pose 23	-261.498	-247.606	28.428	**-271.3**	**-406.537**	**15.728**
Pose 24	-313.036	-308.46	30.765	-252.301	-269.809	28.955
Pose 25	-258.138	-170.901	29.076	-276.666	-393.155	26.094
Pose 26	**-275.951**	**-333.601**	**18.677**	-250.569	-256.222	30.158
Pose 27	-265.896	-264.571	31.53	-268.011	-307.002	25.61
Pose 28	-266.435	-238.621	31.499	-258.863	-298.246	25.678
Pose 29	-282.052	-176.183	28.625	-257.635	-238.621	31.499
Pose 30	-258.863	-298.246	25.678	-253.075	-219.387	25.131

Kappa	PIPER pose energy	PIPER pose score	RMSD	PIPER pose energy	PIPER pose score	RMSD
Pose 1	-281.282	-307.107	28.438	-253.959	-373.522	29.032
Pose 2	-317.854	-385.97	31.672	-267.624	-285.278	28.138
Pose 3	-263.947	-281.689	31.838	-284.543	-378.059	31.52
Pose 4	-264.034	-318.26	31.855	-247.821	-314.659	30.474
Pose 5	-259.699	-349.109	28.919	-253.471	-295.618	29.196
Pose 6	-293.689	-276.164	30.064	-275.512	-246.11	30.394
Pose 7	-270.576	-265.905	29.396	-259.034	-318.26	31.855
Pose 8	-268.675	-152.861	30.756	-260.055	-261.798	32.412
Pose 9	-261.99	-365.964	31.108	-265.742	-353.808	30.149
Pose 10	-322.992	-175.271	30.321	-249.242	-251.592	29.32
Pose 11	-312.337	-296.912	37.1	-260.105	-293.445	27.033
Pose 12	-281.42	-273.528	25.894	**-279.165**	**-393.576**	**17.874**
Pose 13	-273.226	-241.644	29.791	-260.882	-328.221	32.214
Pose 14	-266.405	-193.59	25.506	-284.084	-279.118	26.729
Pose 15	-257.867	-162.112	28.755	-252.815	-340.665	32.712
Pose 16	-263.257	-304.049	32.257	-264.005	-193.59	25.506
Pose 17	-303.523	-264.131	27.995	-257.906	-299.541	26.634
Pose 18	**-267.713**	**-392.977**	**19.661**	-253.771	-175.025	29.852
Pose 19	-257.573	-317.983	31.308	-303.623	-336.459	28.28
Pose 20	-285.904	-267.004	31.203	-257.705	-286.045	25.275
Pose 21	-256.094	-264.492	27.581	-285.221	-206.998	24.242
Pose 22	-306.122	-288.681	28.803	-268.274	-400.627	38.639
Pose 23	-299.066	-161.323	31.616	-270.531	-171.483	25.783
Pose 24	-259.509	-168.621	33.367	-248.327	-289.066	29.859
Pose 25	-268.429	-201.073	33.631	-297.122	-288.681	28.803
Pose 26	-277.079	-195.988	31.205	-250.289	-276.164	30.064
Pose 27	-266.514	-49.318	33.086	-251.494	-194.085	30.222
Pose 28	-260.676	-254.648	28.334	-258.044	-172.865	31.224
Pose 29	-261.552	-207.948	32.712	-253.488	-177.033	31.135
Pose 30	-265.331	-135.285	33.09	-254.517	-223.295	26.295
**Epsilon**	**PIPER pose energy**	**PIPER pose score**	**RMSD**	**PIPER pose energy**	**PIPER pose score**	**RMSD**
Pose 1	-276.304	-266.515	30.04	-266.073	-350.311	28.525
Pose 2	-267.517	-187.545	32.58	-293.708	-339.675	25.724
Pose 3	-305.176	-379.65	26.783	-246.341	-198.502	32.936
Pose 4	-263.026	-183.319	27.852	-250.201	-204.491	27.141
Pose 5	-286.049	-336.668	29.744	-247.996	-266.941	31.697
Pose 6	-262.344	-252.567	27.214	**-254.411**	**-469.888**	**14.809**
Pose 7	-256.445	-231.833	24.355	-289.099	-233.95	28.336
Pose 8	-268.081	-352.393	23.589	-244.282	-384.125	40.2
Pose 9	-289.943	-262.425	28.036	-249.517	-159.58	21.689
Pose 10	-282.977	-122.59	33.32	-283.124	-350.453	30.037
Pose 11	-281.845	-198.497	20.904	-247.877	-238.6	22.638
Pose 12	-266.126	-188.314	23.015	-245.519	-295.817	15.509
Pose 13	-291.956	-151.164	29.044	-266.765	-272.48	16.622

Pose 14	-314.795	-263.895	20.676	-290.181	-220.792	29.736
Pose 15	-286.591	-301.567	27.079	-247.519	-353.129	29.017
Pose 16	-255.369	-316.934	31.36	-246.374	-214.896	15.269
Pose 17	-254.482	-110.412	30.954	-268.407	-382.457	29.016
Pose 18	-262.954	-225.686	27.725	-253.955	-335.19	26.172
Pose 19	-294.181	-220.792	29.736	-251.621	-268.522	27.922
Pose 20	**-269.165**	**-272.48**	**16.622**	-245.352	-300.523	22.982
Pose 21	-275.044	-243.924	36.202	-273.037	-227.761	16.524
Pose 22	-266.393	-112.295	22.949	-278.575	-379.65	26.783
Pose 23	-268.49	-273.234	28.845	-251.38	-348.785	15.409
Pose 24	-269.57	-169.222	19.348	-290.327	-215.66	29.809
Pose 25	-257.912	-66.772	33.157	-255.957	-324.291	24.675
Pose 26	-258.083	-217.844	21.255	-246.811	-401.627	22.714
Pose 27	-273.917	-304.936	18.916	-246.969	-225.654	31.262
Pose 28	-258.915	-193.477	24.085	-245.033	-232.457	30.044
Pose 29	-264.524	-166.242	23.589	-245.815	-220.909	31.708
Pose 30	-262.507	-208.552	29.108	-249.72	-225.443	30.505

**Omicron BA.1**	**PIPER pose energy**	**PIPER pose score**	**RMSD**	**PIPER pose energy**	**PIPER pose score**	**RMSD**
Pose 1	-259.175	-220.062	21.654	-247.021	-220.274	22.635
Pose 2	-315.308	-243.084	27.076	-257.229	-237.425	32.686
Pose 3	-266.588	-248.822	29.863	-282.191	-212.701	28.506
Pose 4	-256.017	-232.97	33.349	-244.039	-312.34	30.755
Pose 5	-277.064	-190.003	28.817	-247.122	-325.191	25.74
Pose 6	-269.941	-413.704	20.935	-271.667	-327.852	29.83
Pose 7	-248.454	-285.774	23.113	-291.992	-347.665	30.106
Pose 8	-272.5	-308.704	27.212	-243.5	-156.04	27.151
Pose 9	-264.461	-224.339	28.986	-270.9	-308.704	27.212
Pose 10	-272.722	-197.193	22.423	-271.504	-169.557	24.602
Pose 11	-291.992	-347.665	30.106	-269.941	-413.704	20.935
Pose 12	-255.843	-266.606	27.49	-264.665	-286.088	27.722
Pose 13	-249.413	-225.209	34.228	-270.578	-307.223	26.903
Pose 14	-249.828	-296.771	29.18	-252.575	-209.332	33.649
Pose 15	-249.24	-270.676	27.514	-248.663	-282.199	27.076
Pose 16	-266.065	-286.088	27.722	-274.284	-339.279	27.595
Pose 17	-250.437	-220.926	30.899	-259.49	-259.294	26.061
Pose 18	**-250.242**	**-226.939**	**20.036**	-267.231	-202.968	30.484
Pose 19	-277.684	-339.279	27.595	-249.524	-197.262	22.605
Pose 20	-255.848	-375.194	25.397	**-257.92**	**-254.487**	**19.803**
Pose 21	-271.667	-327.852	29.83	-241.099	-84.573	30.254
Pose 22	-255.575	-209.332	33.649	-240.946	-283.772	26.354
Pose 23	-253.391	-235.092	26.313	-258.769	-240.859	25.337
Pose 24	-255.799	-116.083	23.981	-258.369	-173.728	30.858
Pose 25	-248.5	-84.573	30.254	-241.678	-214.359	26.151
Pose 26	-250.313	-276.881	23.382	-240.494	-280.882	26.7
Pose 27	-249.586	-216.431	25.521	-241.654	-202.706	21.472
Pose 28	-259.053	-173.207	31.374	-260.35	-166.105	29.392

Pose 29	-248.92	-234.406	27.619	-252.497	-236.178	30.855
Pose 30	-267.535	-146.652	29.343	-243.589	-346.288	26.744
**Omicron JN.1**	**PIPER pose energy**	**PIPER pose score**	**RMSD**	**PIPER pose energy**	**PIPER pose score**	**RMSD**
Pose 1	-309.617	-302.567	32.163	-298.522	-293.757	28.046
Pose 2	-302.922	-293.757	28.046	-248.174	-357.254	23.801
Pose 3	-259.273	-265.253	30.356	-267.688	-322.657	27.857
Pose 4	-342.901	-317.941	28.293	-247.699	-198.878	29.186
Pose 5	-252.589	-223.792	21.632	-256.891	-228.772	31.702
Pose 6	-256.956	-217.708	29.261	-264.131	-415.388	27.969
Pose 7	-249.25	-304.624	26.127	-324.101	-317.941	28.293
Pose 8	-290.833	-312.659	28.261	-250.516	-248.582	30.005
Pose 9	-301.016	-116.137	32.429	-254.494	-222.592	22.438
Pose 10	**-266.662**	**-170.247**	**21.548**	-252.184	-437.827	27.27
Pose 11	-258.631	-232	37.379	-258.18	-354.314	31.282
Pose 12	-254.495	-222.592	22.438	-237.02	-154.045	27.372
Pose 13	-257.432	-247.645	34.086	-254.892	-295.922	34.473
Pose 14	-256.094	-198.887	29.526	-240.201	-324.078	31.843
Pose 15	-264.135	-261.92	28.046	-242.314	-191.143	26.748
Pose 16	-253.15	-211.632	27.557	-239.376	-371.035	27.029
Pose 17	-264.132	-415.388	27.969	-250.693	-198.887	29.526
Pose 18	-261.604	-220.645	33.593	-279.216	-116.137	32.429
Pose 19	-253.696	-241.086	23.305	-266.993	-302.209	33.588
Pose 20	-248.956	-332.295	29.258	-236.82	-223.613	37.724
Pose 21	-258.18	-354.314	31.282	**-252.605**	**-250.939**	**20.979**
Pose 22	-271.675	-212.183	29.012	-253.703	-279.662	32.071
Pose 23	-266.993	-302.209	33.588	-238.456	-345.208	25.045
Pose 24	-249.558	-248.757	26.922	-240.37	-262.601	27.813
Pose 25	-252.184	-437.827	27.27	-243.92	-272.331	26.975
Pose 26	-244.977	-288.883	22.899	-236.535	-358.167	25.811
Pose 27	-245.641	-317.356	31.919	-236.418	-310.52	30.63
Pose 28	-250.737	-252.177	28.015	-242.475	-352.223	26.839
Pose 29	-251.654	-262.113	24.459	-244.078	-259.833	27.87
Pose 30	-244.792	-234.106	34.962	-245.736	-252.177	28.015

Table S14. Sequences of 2B04 antibody and mutant's RBDs.

**Antibody Heavy Chain (H)**
QVQLKQSGPGLVAPSQSLSITCTVSGFSLINYAISWVRQPPGKGLGVIWTGGGTNYNSALKSRSISKDNSKSQVFL KMNS LQTDDTARYYCARKDYYGRY YGMDYWGQGTSVTVS
**Antibody Light Chain (L)**
QAVVTQESALTTSPGETVTLTCRSSTGAVTTSNYANWVQEKPDHLFTGLIGGTNNRAPGVPARFSGSLIGDKAAL T TGAQTEDEAIFYCALWYN NHWVFGGGTKLTVL
**RBD (Wild Type)**
TNLCPFGEVFNATRFASVYAWNRKRISNCVADYSVLYNSASFSTFKCYGVSPTKLNDLCFTNVYADSFVIRGDEV RQIAPGQTGKIADYNYKLPDDFTGCVIAWNSNNLDSKVGGNYNYLYRLFRKSNLKPFERDISTEIYQAGSTPCNG VEGFNCYFPLQSYGFQPTNGVGYQPYRVVVLSFELLHAPATVCG

**RBD (Alpha)**
TNLCPFGEVFNATRFASVYAWNRKRISNCVADYSVLYNSASFSTFKCYGVSPTKLNDLCFTNVYADSFVIRGDEV
RQIAPGQTGKIADYNYKLPDDFTGCVIAWNSNNLDSKVGGNYNYLYRLFRKSNLKPFERDISTEIYQAGSTPCNG
VEGFNCYFPLQSYGFQPTYGVGYQPYRVVVLSFELLHAPATVCG
**RBD (Beta)**
TNLCPFGEVFNATRFASVYAWNRKRISNCVADYSVLYNSASFSTFKCYGVSPTKLNDLCFTNVYADSFVIRGDEV
RQIAPGQTGNIADYNYKLPDDFTGCVIAWNSNNLDSKVGGNYNYLYRLFRKSNLKPFERDISTEIYQAGSTPCNG
VKGFNCYFPLQSYGFQPTYGVGYQPYRVVVLSFELLHAPATVCG
**RBD (Gamma)**
TNLCPFGEVFNATRFASVYAWNRKRISNCVADYSVLYNSASFSTFKCYGVSPTKLNDLCFTNVYADSFVIRGDEV
RQIAPGQTGTIADYNYKLPDDFTGCVIAWNSNNLDSKVGGNYNYLYRLFRKSNLKPFERDISTEIYQAGSTPCNG
VKGFNCYFPLQSYGFQPTYGVGYQPYRVVVLSFELLHAPATVCG
**RBD (Delta)**
TNLCPFGEVFNATRFASVYAWNRKRISNCVADYSVLYNSASFSTFKCYGVSPTKLNDLCFTNVYADSFVIRGDEV
RQIAPGQTGKIADYNYKLPDDFTGCVIAWNSNNLDSKVGGNYNYRYRLFRKSNLKPFERDISTEIYQAGSKPCNG
VEGFNCYFPLQSYGFQPTNGVGYQPYRVVVLSFELLHAPATVCG
**RBD (Kappa)**
TNLCPFGEVFNATRFASVYAWNRKRISNCVADYSVLYNSASFSTFKCYGVSPTKLNDLCFTNVYADSFVIRGDEV
RQIAPGQTGKIADYNYKLPDDFTGCVIAWNSNNLDSKVGGNYNYRYRLFRKSNLKPFERDISTEIYQAGSTPCNG
VQGFNCYFPLQSYGFQPTNGVGYQPYRVVVLSFELLHAPATVCG
**RBD (Epsilon)**
TNLCPFGEVFNATRFASVYAWNRKRISNCVADYSVLYNSASFSTFKCYGVSPTKLNDLCFTNVYADSFVIRGDEV
RQIAPGQTGKIADYNYKLPDDFTGCVIAWNSNNLDSKVGGNYNYRYRLFRKSNLKPFERDISTEIYQAGSTPCNG
VEGFNCYFPLQSYGFQPTNGVGYQPYRVVVLSFELLHAPATVCG
**RBD (Omicron BA.1)**
TNLCPFDEVFNATRFASVYAWNRKRISNCVADYSVLYNLAPFFTFKCYGVSPTKLNDLCFTNVYADSFVIRGDEV
RQIAPGQTGNIADYNYKLPDDFTGCVIAWNSNKLDSKVSGNYNYLYRLFRKSNLKPFERDISTEIYQAGNKPCNG
VAGFNCYFPLRSYSFRPTYGVGHQPYRVVVLSFELLHAPATVCG
**RBD (Omicron JN.1)**
TNLCPFHEVFNATRFASVYAWNRTRISNCVADYSVLYNFAPFFAFKCYGVSPTKLNDLCFTNVYADSFVIKGNEV
SQIAPGQTGNIADYNYKLPDDFTGCVIAWNSNKLDSKHSGNYDYWYRSFRKSKLKPFERDISTEIYQAGNKPCK
GVKGPNCYFPLQSYGFRPTYGVGHQPYRVVVLSFELLHAPATVCG
